# Gene context drift identifies drug targets to mitigate cancer treatment resistance

**DOI:** 10.1016/j.ccell.2025.06.005

**Published:** 2025-06-26

**Authors:** Amir Jassim, Birgit V. Nimmervoll, Sabrina Terranova, Erica Nathan, Linda Hu, Jessica T. Taylor, Katherine E. Masih, Lisa Ruff, Matilde Duarte, Elizabeth Cooper, Gunjan Katyal, Melika Akhbari, Reuben J. Gilbertson, Jennifer C. Coleman, Joseph S. Toker, Colton Terhune, Gabriel Balmus, Stephen P. Jackson, Hailong Liu, Tao Jiang, Michael D. Taylor, Kui Hua, Jean E. Abraham, Mariella G. Filbin, Anthony Hill, Anarita Patrizi, Neil Dani, Aviv Regev, Maria K. Lehtinen, Richard J. Gilbertson

**Affiliations:** 1 Cancer Research UK Cambridge Institute, University of Cambridge, Li Ka Shing Centre, Robinson Way, Cambridge CB2 0RE, UK; 2 Genetics Branch, Center for Cancer Research, National Cancer Institute, NIH, Bethseda, MD 20892, USA; 3 UK Dementia Research Institute at the University of Cambridge and Department of Clinical Neurosciences, University of Cambridge, Cambridge Biomedical Campus, Cambridge CB2 0AH, UK; 4 Department of Molecular Neuroscience, Transylvanian Institute of Neuroscience, 400191 Cluj-Napoca, Romania; 5 Department of Radiotherapy, Beijing Tiantan Hospital Capital Medical University, Beijing 100070, China; 6 Department of Pediatric Neurosurgery, Beijing Tiantan Hospital Capital Medical University, Beijing 100070, China; 7 Texas Children’s Cancer and Hematology Center, Houston, TX 77030, USA; 8 Department of Pediatrics, Hematology/Oncology, Baylor College of Medicine, Houston, TX 77030, USA; 9 Department of Neurosurgery, Baylor College of Medicine, Houston, TX 77030, USA; 10 Department of Neurosurgery, Texas Children’s Hospital, Houston, TX 77030, USA; 11 Dan L Duncan Comprehensive Cancer Center, Baylor College of Medicine, Houston, TX 77030, USA; 12 Department of Oncology, University of Cambridge, Box 197 Cambridge Biomedical Campus, Cambridge CB2 0XZ, UK; 13 Precision Breast Cancer Institute, Box 197 Cambridge Biomedical Campus, Cambridge CB2 0QQ, UK; 14 Boston Children’s Hospital, 300 Longwood Avenue, Boston, MA 02115, USA; 15 Schaller Research Group, German Cancer Research Center (DKFZ), 69120 Heidelberg, Germany; 16 Department of Cell and Developmental Biology, Vanderbilt School of Medicine, Nashville, TN 37232, USA; 17 Broad Institute of MIT and Harvard, Cambridge, MA, USA

## Abstract

Cancer treatment often fails because combinations of different therapies evoke complex resistance mechanisms that are hard to predict. We introduce *RE*sistance through *CO*ntext *DR*ift (RECODR): a computational pipeline that combines co-expression graph networks of single-cell RNA sequencing profiles with a graph-embedding approach to measure changes in gene co-expression context during cancer treatment. RECODR is based on the idea that gene co-expression context, rather than expression level alone, reveals important information about treatment resistance. Analysis of tumors treated in preclinical and clinical trials using RECODR unmasked resistance mechanisms –invisible to existing computational approaches– enabling the design of highly effective combination treatments for mice with choroid plexus carcinoma, and the prediction of potential new treatments for patients with medulloblastoma and triple-negative breast cancer. Thus, RECODR may unravel the complexity of cancer treatment resistance by detecting context-specific changes in gene interactions that determine the resistant phenotype.

## Graphical abstract

**Graphical abstract:**
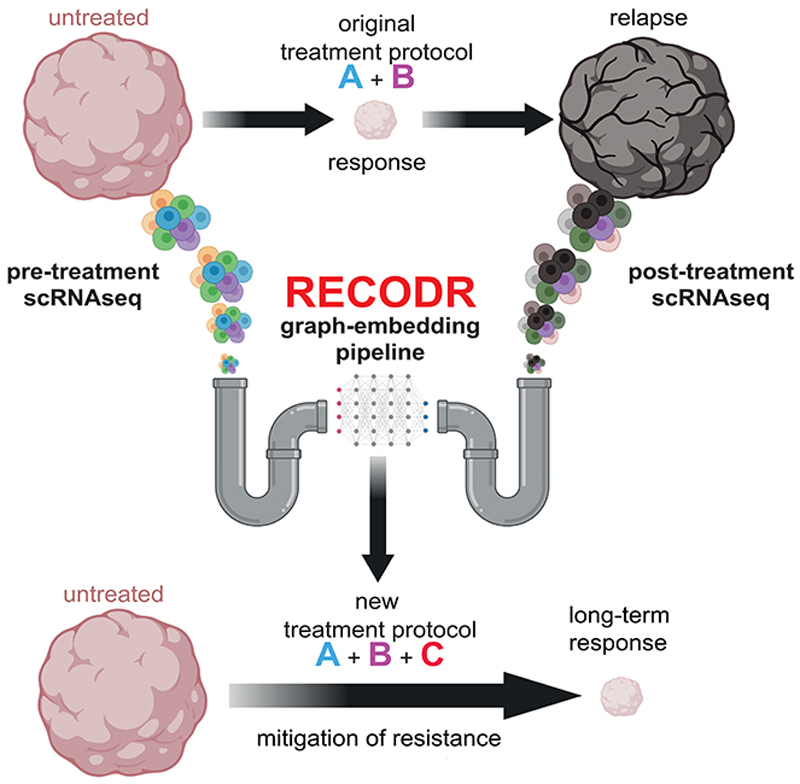

## Introduction

Cancers comprise heterogeneous populations of normal and malignant cells that respond differently to treatment, producing complex and dynamic patterns of efficacy and resistance that are hard to predict.^1,2^ Cancers also vary in their dependence on therapeutic targets. Different tumors can be promoted or suppressed by the same mutation, and cancers expressing similar levels of a drug target can vary in their response to small molecule inhibitors.^3–5^ In the absence of better ways to predict treatment response, cancer therapy has evolved to include combinations of different treatments in the hope they will evoke non-overlapping patterns of efficacy and resistance.^6–8^ But combination therapy also often fails, most likely because it does not account for the complex biology that underpins treatment resistance.^9^ Consequently, 30–50% of patients with cancer fail first-line therapy,^10,11^ the majority eventually dying of their disease.^12–14^

Advanced computation is being used increasingly in biomedicine to solve intractable problems. This includes the use of graph networks (GNs)^15–17^ and machine learning^18,19^ to interrogate large cancer datasets, such as cancer transcriptomes, with the goal of designing more effective treatments. Although these approaches can map gene expression networks in cancer, they typically assign meaning to these networks by comparing them to gene sets detected in other, potentially less relevant, contexts and by employing relatively simple metrics to predict therapeutic targets e.g., gene expression level. None of these techniques have been widely established.

Here, we introduce a natural language processing (NLP)-inspired approach to design effective combination cancer treatments. NLP was originally developed to “read” large bodies of text, generating powerful representations of words with embedding vectors, thereby learning the context in which words are typically found. We reasoned that such word-embedding techniques could be repurposed for gene co-expression data, enabling gene co-expression relationships to be charted during cancer development and treatment resistance. We hypothesized that genes undergoing the greatest change in transcriptome context contribute the most to disease progression and treatment resistance and are therefore effective treatment targets. By executing this approach through a single computational pipeline that we term *RE*sistance through *CO*ntext *DR*ift (RECODR; Figure S1), we mapped changes in gene context during tumorigenesis and treatment resistance in a mouse model of a childhood brain tumor and in human medulloblastomas and triple-negative breast cancers (TNBC) during therapy. RECODR unmasked resistance mechanisms–invisible to conventional genomic and advanced computational approaches–that enabled the design of highly effective combination treatments for the mouse, and the prediction of potential new treatments for the patients with medulloblastoma and TNBC in the clinic.

## Results

### RECODR-prediction of primary tumor vulnerabilities

Choroid plexus carcinoma (CPC) is a rare brain tumor of very young children that arises in the embryonic choroid plexus (CP).^20,21^ No randomized clinical trials have been completed among children with CPC and no molecular targeted treatments for the disease have been identified.^22^ *TP53-*mutant CPC is particularly aggressive: most children with this disease die within five years of diagnosis.

We previously generated a genetically engineered mouse model of CPC that recapitulates the histology, transcriptome, and DNA copy number variations of the human disease.^23,24^ To understand how CP is transformed to CPC, we generated single-cell RNA sequencing (scRNA-seq) profiles of 2,982 CPC cells and compared these with previously published CP scRNA-seq profiles^25^ using RECODR. scRNA-seq profiles of CPC were validated as tumor cell-derived by stringent fluorescence-activated cell sorting (FACS)—since our CPC is engineered to express yellow and red-fluorescence protein (YFP^+^/ RFP^+^; Figures S2A and S2B)–and by inferred chromosomal copy number and structural variations that we confirmed using spectral karyotyping and fluorescence *in situ* hybridization (Figures S2C–S2G).

**Figure 1:**
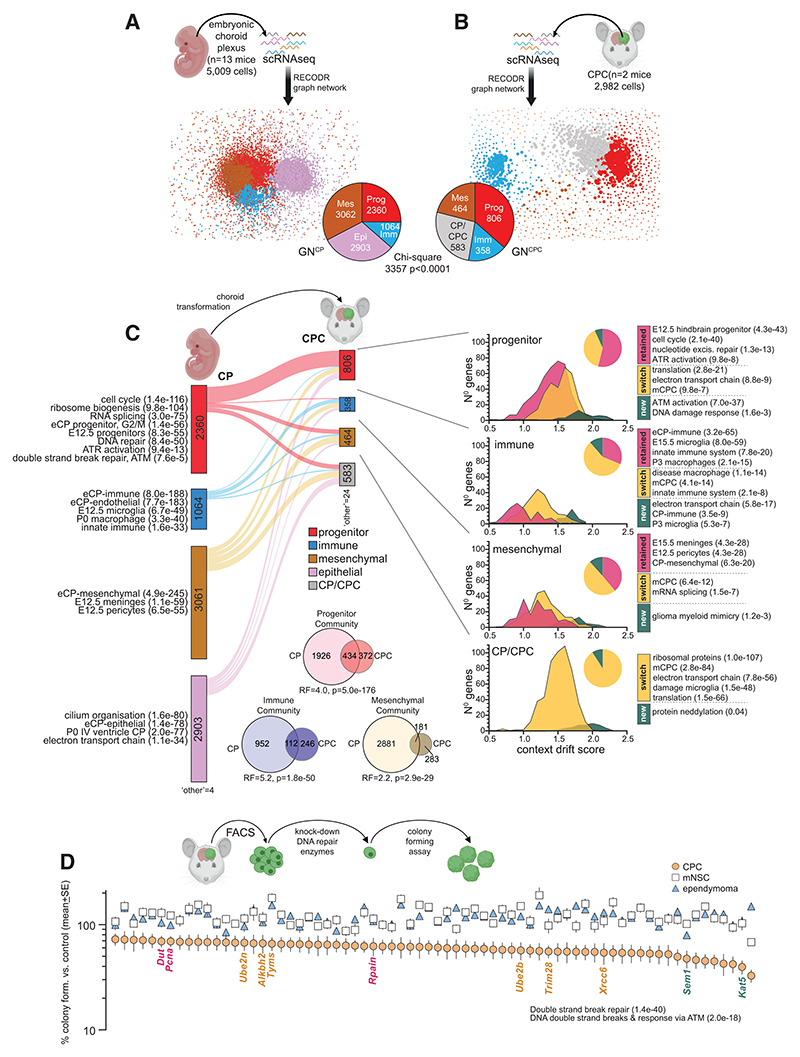
Gene context drift during CPC tumorigenesis RECODR generated graph networks (GNs) of scRNA-seq profiles of (A) embryonic choroid plexus (CP) and (B) untreated CPC. Pie charts indicate the number of genes in progenitor (Prog), immune (Imm), mesenchymal (Mes), epithelial (Epi) or CP/CPC communities. Chi-square reports differences in gene numbers in communities between (GNs). (C) Alluvial plot of gene locations between communities in the GN^CP^ and GN^CPC^. Enriched pathways in relevant gene groups are shown with false discovery rates. Frequency plots of gene-types reported in the main text are shown right. The associated pie charts report the proportion of “new”, “switched”, and “retained” genes in the corresponding communities. Venn diagrams bottom show overlap in genes in the indicated communities between GN^CP^ and GN^CPC^ with representation factor (RF) and associated *p*-value for overlap. (D) Cartoon illustrates the approach to calculate the percent colony formation by fluorescence-activated cell sorting (FACS) isolated CPC, mouse neural stem cells (mNSCs) and mouse *RTBDN*-driven ependymoma cells reported in the graph following shRNA silencing of the indicated DNA repair transcript relative to matched-control transduced cells (circles are average, error bars standard error). Only genes demonstrating significant (*p* < 0.05) growth suppression in CPC cells are shown. New (green), switched (brown) and retained (red) genes also present on the GN^CPC^ are highlighted. Enriched pathways in CPC dependency genes are shown.

RECODR organized genes within CP and CPC scRNA-seq profiles into co-expression GNs in which genes with correlated expression, and potentially related function, were clustered together into four distinct communities (Figures 1A and 1B; Table S1). In keeping with its origin, 91% (*n* = 2,030/2,235) of all genes in the CPC GN (GN^CPC^) overlapped with those in the CP GN (GN^CP^; representation factor for overlap, *p* = 2.4e-243) and three of the four communities in both GNs were enriched for genes associated with progenitor, immune, or mesenchymal cells (Table S1). Around one-third (*n* = 727/2,030) of genes common to both GNs were located in the same community type–we termed these “retained” genes since they likely mediated similar functions in both the normal parent and malignant daughter tissue (Figure 1C). RECODR analysis of four human CPC single nuclear RNAseq profiles produced a similar GN structure (Figure S3A and Table S1). Therefore, like other childhood cancers^26^ CPC appears to retain features of the embryonic parent tissue, suggesting stalled development contributes to its pathogenesis.

But RECODR also detected striking differences between the GN^CP^ and GN^CPC^ (Figure 1C; Table S2). In keeping with a role for stalled development in CPC tumorigenesis, a large community of genes associated with ciliated epithelium on the GN^CP^ was replaced on the GN^CPC^ by a community of genes associated with embryonic and malignant CP (CP/CPC community). Furthermore, two-thirds (*n* = 1,303/2,030) of genes that persisted on the GN^CPC^ from the GN^CP^ switched community type during transformation, suggesting the functional context of these genes was altered in tumors—we termed these “switched” type genes. Nine percent (*n* = 205/2,235) of genes were newly recruited to the GN^CPC^ during transformation (Figure 1C and Table S2).

To gain further insights into how each gene changed its transcriptome context during transformation, we deployed the graph-embedding approach within RECODR to learn and compare the context of each gene between the GN^CP^ and GN^CPC^ (Figure S1). First, using the Node2Vec^27^ algorithm, RECODR took 200 random walks from each gene across the GN^CP^ and GN^CPC^ creating 100,000s of walks. RECODR then fed these walks into the Word2Vec^28^ neural network to learn the context of each gene in each GN. RECODR then calculated a *context drift score* for each gene by summating four independent metrics (STAR Methods): (1) *neighbor numbers score* – the number of new genes directly connected to the index gene on the GN^CPC^ relative to the GN^CP^ normalized to the total number of genes in the GN^CPC^; (2) *graph reach score* – the number of immediate neighbors of the index gene on the GN^CPC^ plus the unique connections of these direct neighbors normalized to the total number of genes on the GN^CPC^; (3) *neighbor cosine score* – the average transcriptome position shift determined as the average of all cosine vector scores of genes directly connected to the index gene; and (4) *index gene cosine score* – the transcriptome position shift determined as a cosine vector score of the index gene.

Genes with the highest context drift scores were located within the progenitor and CP/CPC communities, suggesting these played the greatest role in CPC tumorigenesis (Figures 1C, S3B, and S3C; Table S2). Genes “newly” recruited to the GN^CPC^ progenitor community displayed the highest context drift and were especially enriched for regulators of ataxia telangiectasia mutated (ATM; FDR = 7.0e-37; Table S2), including *Kat5*.^29^ Genes “retained” by the progenitor community were enriched for regulators of embryonic neural progenitors (FDR = 4.3e-43) and various DNA repair pathways, pinpointing these as potentially important in the maintenance of CPC (Table S2). Genes that “switched” community type to the GN^CPC^ progenitor community were enriched for regulators of protein and energy production that are well recognized features of malignancy.^30,31^

Since regulators of DNA repair displayed high levels of context drift during transformation of the CP, we looked to see if DNA repair was important for maintaining CPC. First, we measured the impact of knocking down 227 DNA repair genes on CPC clonogenic colony formation *in vitro*. Seventy of these genes were required for efficient colony formation (Figure 1D and Table S3). These required genes were highly enriched for regulators of double-strand break repair via ATM, including *Kat5*–the second most required gene—supporting the notion that gene context drift detected by RECODR identifies genes important in transformation.

To test more directly if ATM is a therapeutic vulnerability of CPC, we treated mice with CPC with four different regimens of the blood-brain barrier penetrating ATM inhibitor AZD1390 (Figures S4A–S4C).^32,33^ We compared this treatment to various doses and schedules of fractionated radiotherapy that is used to treat older children and adults with CPC (Figures S4D–S4G). Two AZD1390 protocols significantly suppressed CPC growth, one of which also extended mouse survival. To our knowledge, this is the first report of single agent ATM inhibitor efficacy in cancer. Like human CPC, the mouse disease was also sensitive to radiation therapy.

To benchmark RECODR’s prediction of ATM as a therapeutic vulnerability, we also analyzed CP and CPC scRNA-seq profiles using differential gene expression analysis and three more advanced computational approaches that were developed to pinpoint key signal pathways and/or therapeutic targets from scRNA-seq data (see STAR Methods): Weighted Gene Co-expression Network Analysis (WGCNA),^34^ Differential Co-expression Analysis (diffcoexp; https://github.com/hidelab/diffcoexp) and single-cell DRUG (scDRUG).^35^ None of these approaches identified ATM as a high-priority target in CPC (Tables S4 and S5).

### RECODR-predicts monotherapy resistance

**Figure 2:**
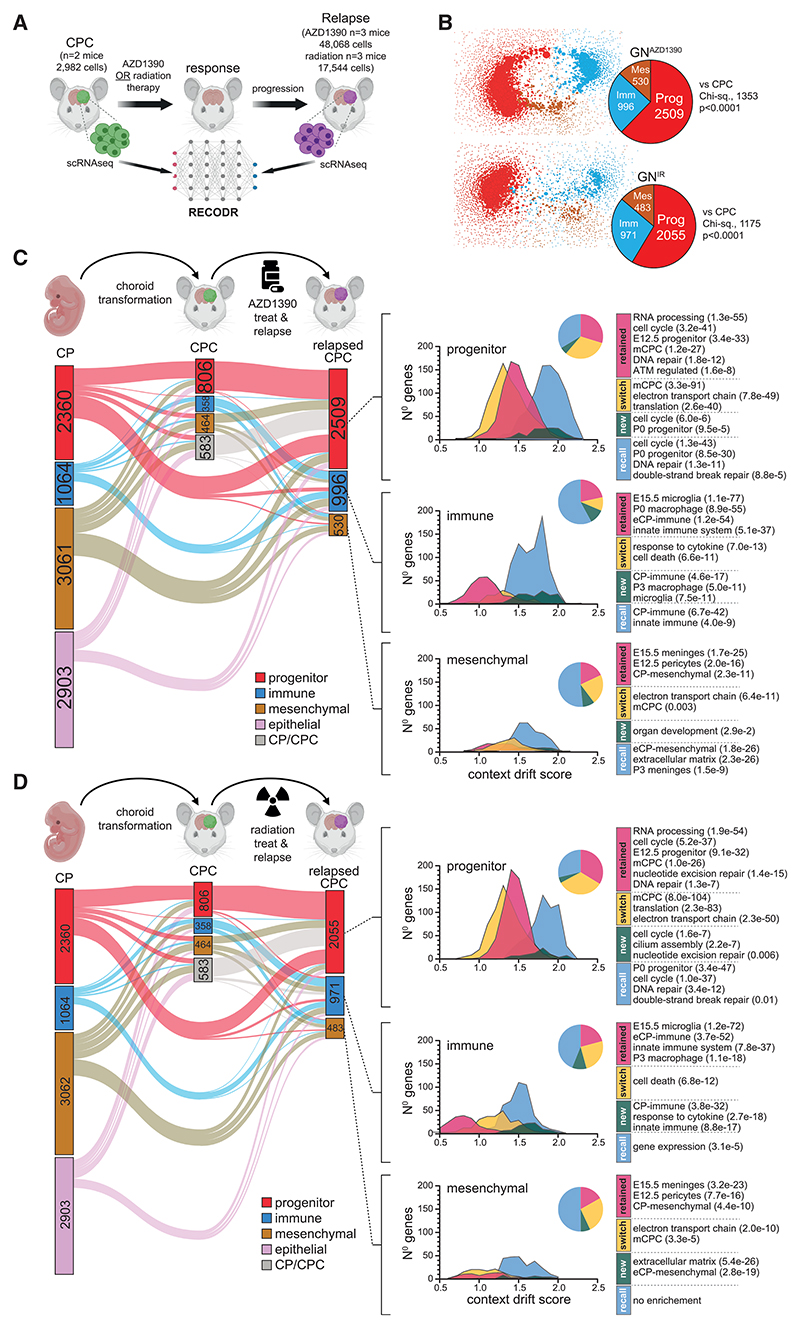
Gene context drift during monotherapy resistance (A) Cartoon depicting the analysis of scRNA-seq profiles by RECODR during monotherapy resistance. (B) RECODR generated graph networks (GNs) of scRNA-seq profiles of CPC that relapsed during AZD1390 (top) or irradiation (bottom) therapy. Venn diagrams report numbers of genes in the indicated GN communities. Chi-square reports differences in gene numbers in communities between relapsed CPC and control CPC GNs. Alluvial plots of gene communities in (C) GN^CP^, GN^CPC^, GN^AZD1390^, and (D) GN^CP^, GN^CPC^, GN^IR^. Enriched pathways in relevant gene groups are shown with false discovery rates. Frequency plots of gene-types reported in the main text are shown right in each. The associated pie charts report the proportion of “recalled”, “new”, “switched”, and “retained” genes in the corresponding communities.

Like most human cancers treated with monotherapy, all CPCs treated with AZD1390 or radiation ultimately relapsed (Figures S4F and S4G). Therefore, we looked to see if RECODR could identify genes that changed their context during monotherapy resistance since we hypothesized these contributed to treatment failure. To do this, we used RECODR to compare scRNA-seq profiles of untreated CPC with those generated from tumors that had failed AZD1390 (*n* = 48,068 cells) or radiation (*n* = 17,544 cells) treatment (Figure 2A).

Despite their different modes of action, RECODR generated very similar GNs of AZD1390-(GN^AZD1390^) and radiation-relapsed (GN^IR^) tumors that were strikingly different from the GN^CPC^, suggesting tumors resisted these treatments through similar mechanisms (Figure 2B; Tables S1 and S2). Both relapsed GNs contained progenitor communities that were two to three-times larger than that of the GN^CPC^. Like the transcriptome changes detected by RECODR during the transformation of CP to CPC, genes in these progenitor communities had undergone the greatest context drift during treatment resistance, suggesting they were particularly important in resisting AZD1390 or radiation monotherapy (Figure S5A and Table S2). Remarkably, around a third of these relapsed progenitor community genes were absent from the untreated GN^CPC^, but present in the embryonic GN^CP^, suggesting that these genes mediated functions that had been “recalled” from the ancestral embryonic tissue during treatment resistance (Figures 2C and 2D; Table S2). “Recalled” genes accounted for 42% (*n* = 1,696/4,035) and 34% (*n* = 1,261/3,509) of all genes on the GN^AZD1390^ and GN^IR^, respectively. These data are in keeping with observations that primitive fetal programs reemerge during treatment resistance in cancer.^5,36^ “Recalled” genes further enriched relapsed progenitor communities with regulators of the cell cycle (e.g., *Cdk6, Cdk7, Ccna2, Ccnb2*, and *Ccnd3*) and DNA double-strand break repair (e.g., *Parp1, Rad50, Rad51*) and underwent the highest context drift of any genes in relapsed tumors (Figures 2C, 2D, and S5B; Table S2). The remaining genes within relapsed progenitor communities were “retained” from the GN^CPC^ progenitor community, or “switched” from other community types in the GN^CPC^. Surprisingly few genes were “new” to the GN^AZD1390^ or GN^IR^.

Thus, RECODR predicted that CPCs resisted AZD1390 and radiation monotherapy by retaining cell proliferation and DNA repair activity present in untreated tumors, and enhancing these functions further by recalling capabilities active in embryonic CP. Indeed, nuclear γH2AX expression that marks DNA double-strand breaks decreased significantly in monotherapy resistant CPCs indicating increased DNA repair capacity in resistant tumors (Figures S5C and S5D).

**Figure 3:**
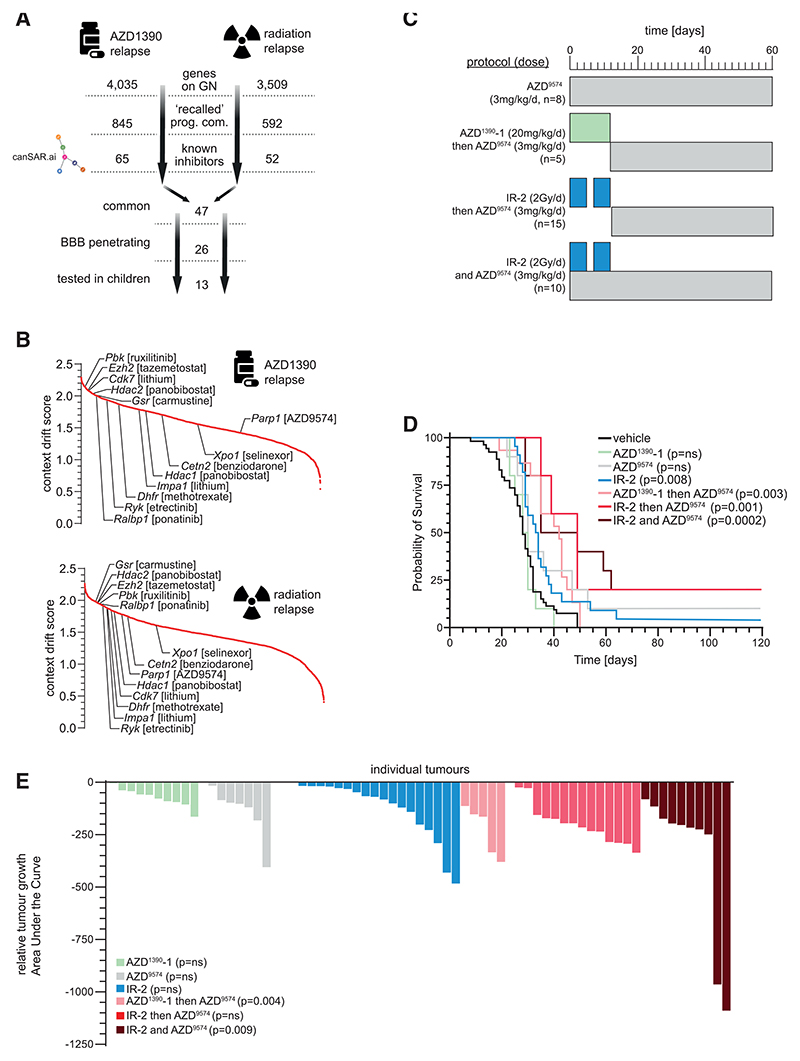
RECODR identification of drug targets in monotherapy resistant tumors (A) Triage of candidate gene targets to mitigate both AZD1390 and radiation monotherapy resistance (prog. com = progenitor community; BBB = blood brain barrier). (B) Ranked context drift scores of all genes on the GN^AZD1390^ (top) and GN^IR^ (bottom). Exemplar target genes emerging from triage process are shown with associated inhibitors in square brackets. (C) Preclinical trial designs to mitigate monotherapy resistance (n = number of mice enrolled in each arm). (D) Survival curves for the corresponding preclinical treatment protocols in (C). Monotherapy treatments are shown for comparison (*p* values report the Log-Rank statistic relative to control treatment). (E) Waterfall plots reporting tumor growth suppression of each individual mouse tumor over time during the indicated treatment. Bars report the total area under the curve of tumor growth over time measured by weekly tumor bioluminescence relative to control treated growth (*p* values record significant difference in growth relative to controls by Mann–Whitney test).

To test if genes that changed their context the most during AZD1390 and radiation resistance drove treatment failure, we looked for inhibitors of genes displaying the highest context drift scores between the GN^CPC^ and relapsed GNs. To select targets and matched inhibitors with the greatest probability of translation, we triaged the 4,035 and 3,509 genes on the GN^AZD1390^ and GN^IR^, respectively, for those: (1) “recalled” to progenitor communities on both the GN^AZD1390^ and GN^IR^–since these had the highest context drift scores; (3) with clinically proven inhibitors known to cross the blood-brain barrier; and (3) with inhibitor classes previously tested in children (Figure 3A and Table S2). The 13 targets emerging from this triage process included *Ezh2* that was recently suggested to mediate histone methylation and repression of *ZIC4* in human CPC,^37^ and *Dhfr* that has been targeted successfully with methotrexate among children with *TP53*-mutant CPC^38^ (Figure 3B). Indeed, DHFR protein expression was increased significantly in CPC cells following AZD1390 or radiation resistance (Figures S5E and S5F).

The remaining 11 triaged targets included *Parp1* for which a new inhibitor (AZD9574) was recently designed to cross the blood-brain barrier to treat brain tumors. Therefore, we tested if PARP1 inhibition could mitigate AZD1390 or radiation monotherapy resistance by dosing CPC-bearing mice with AZD9574 following, and/or during, AZD1390 or radiation treatment. Toxicity precluded simultaneous dosing of mice with AZD1390 and AZD9574. We compared this treatment to therapy with AZD9574 alone that RECODR predicted would be inactive against untreated CPC: *Parp1* was absent from the GN^CPC^ (Figure 3C; Tables S1 and S2). In keeping with these predictions, AZD9574 monotherapy was ineffective against previously untreated CPC but significantly enhanced the efficacy of either AZD1390 or radiation alone (Figures 3D and 3E).

Benchmarking again demonstrated that RECODR out-performed other target prediction approaches: *Parp1* was not differentially expressed, or differentially co-expressed (determined by *diffcoexp*), among untreated or relapsed CPC cells; scDRUG predicted PARP1 to be an equally effective target in previously untreated, AZD1390-and radiation-relapsed tumors, likely because the model underlying scDrug is trained on cancer cell lines *in vitro* that are unlikely to capture the full complexity of transcriptomic structures *in vivo*; and WGCNA deprioritized PARP1 in radiation-relapsed tumors relative to RECODR (Tables S4 and S5).

### RECODR-predicts combination therapy resistance

Although adding AZD9574 to AZD1390 or radiation treatment increased therapeutic response, all mice receiving AZD1390 and AZD9574 relapsed, and only 20% of mice receiving AZD9574 and radiation achieved long-term survival. Therefore, we looked to see if RECODR might help design more effective combination treatment strategies. We focused on combinations of AZD1390 and radiation that are being tested in the clinic (NCT03423628).

**Figure 4:**
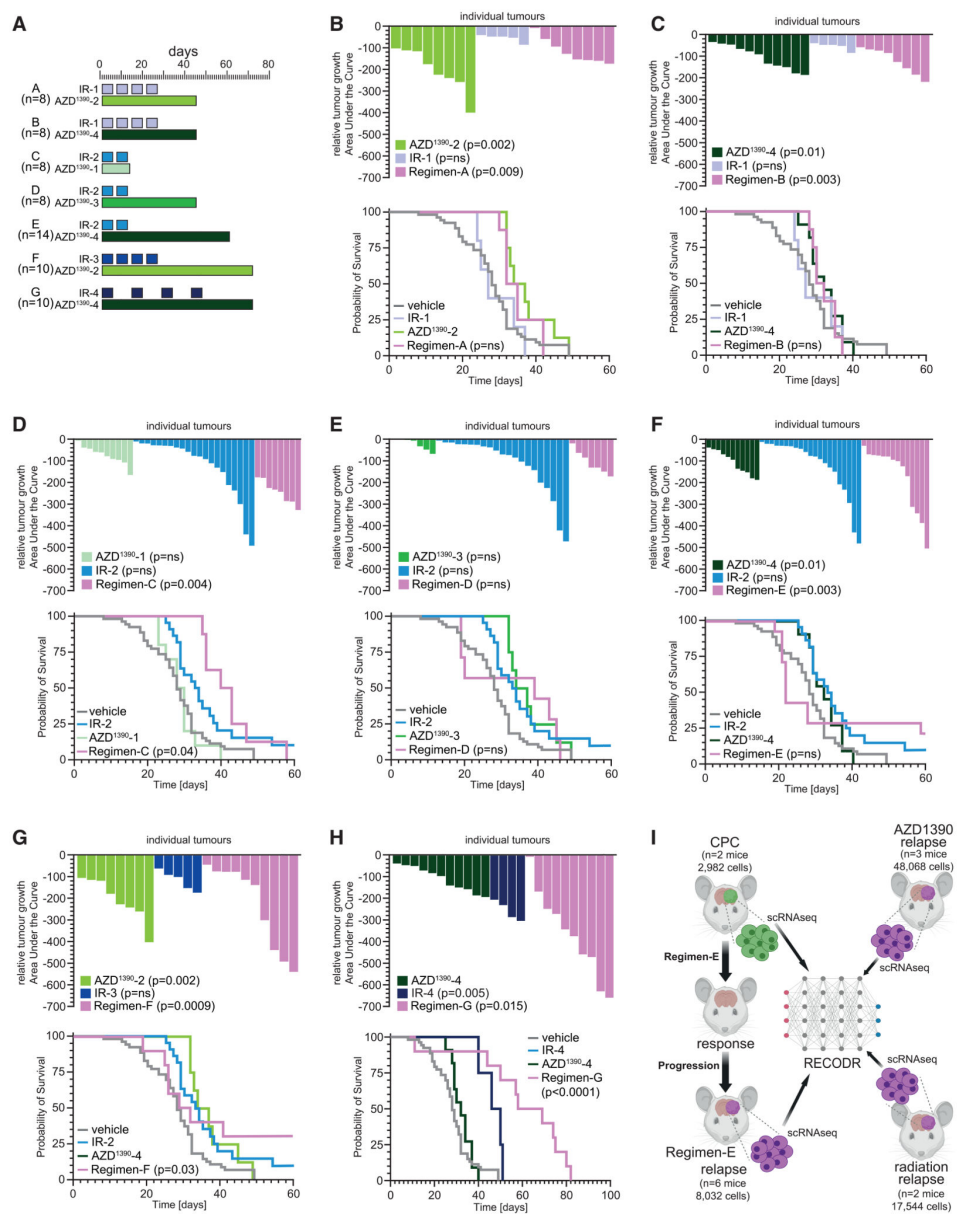
Combination AZD1390 and irradiation treatment regimens (A) Preclinical trial designs to mitigate monotherapy resistance (n = number of mice enrolled in each arm). (B–H) Waterfall plots (top in each) reporting the growth suppression of each individual tumor over time during the indicated treatment. Bars report the total area under the curve of tumor growth over time measured by weekly tumor bioluminescence relative to control treated growth (*p* values record significant difference in growth relative to controls by Mann–Whitney test). Survival curves (bottom in each) report survival for the corresponding preclinical treatment protocols. Monotherapy treatments are shown for comparison (*p* values report the Log-Rank statistic relative to control treatment). (I) Cartoon depicting the analysis of scRNA-seq profiles by RECODR during monotherapy and combination treatment resistance.

We randomized 65 mice with CPC to one of seven different AZD1390 and radiation combination protocols ranging from low to high intensity (Regimens A-G; Figure 4A). Five regimens (B, C, E, F and G) inhibited CPC growth and/or extended mouse survival significantly more than monotherapy alone, although all protocols eventually failed (Figures 4B–4H).

**Figure 5:**
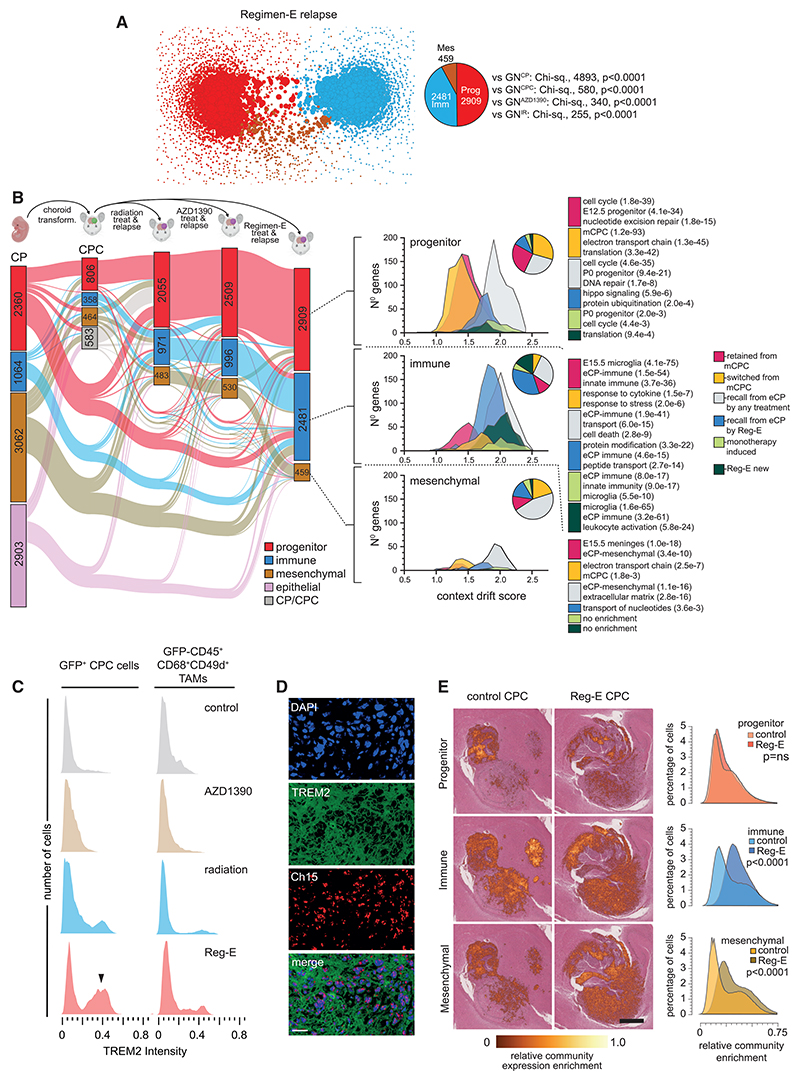
Gene context drift during Regimen-E combination treatment resistance (A) RECODR generated graph networks (GN) of scRNA-seq profiles of CPC that relapsed during Regimen-E treatment. Venn diagram reports number of genes in the indicated community. Chi-square reports differences in gene numbers in communities between Regimen-E relapsed CPC and the other indicated GNs. (B) Alluvial plot of gene communities in GN^CP^, GN^CPC^, GN^AZD1390^, GN^IR^ and GN^Reg-E^. Enriched pathways in relevant gene groups are shown with false discovery rates. Frequency plots of gene-types reported in the main text are shown right in each. The associated pie charts report the proportion of gene-types (key far right) in the corresponding communities. (C) Frequency plots of TREM2 immunofluorescence across different conditions and control (untreated CPC) in GFP^+^ CPC cells or GFP^-^/CD45^+^/CD69^+^/CD49^+^ tumor associated macrophages (TAMs). Arrow indicates subpopulation of TREM2 high expressing CPC cells. (D) Representative concurrent TREM2 immunofluorescence and chromosome 15 FISH of Regimen-E treated CPC. Scale bar = 20μm. (E) Enrichment of expression of the indicated community detected by Visium HD spatial transcriptomics analysis of control and Regimen-E relapsed CPC (scale bar = 1μm). Frequency plots of relative enrichment of expression of the indicated community in CPC following the indicated treatment detected by Visium HD (*p* value, Mann–Whitney test).

To identify genes that might have driven resistance to combination AZD1390 and radiation treatment, we generated 8,032 scRNA-seq profiles from Regimen-E relapsed tumours–a combination similar to that used in the clinic–and compared these with GNs of CP, untreated CPC, and monotherapy relapsed tumors using RECODR (Figure 4I). The Regimen-E relapsed CPC GN (GN^Reg-E^) contained a markedly expanded immune community relative to that of either monotherapy relapsed GN or untreated GN^CPC^ (Figures 5A and 5B; Table S1). RECODR also assigned the highest context drift scores to genes within the GN^Reg-E^ immune community (Figures S5G and S5H; Table S2). This expanded immune community included 82% (*n* = 390/475) and 53% (*n* = 1,538/2,912) of all genes that were “new” to the GN^Reg-E^ or “recalled” from the embryonic GN^CP^, respectively. These genes enriched the GN^Reg-E^ for regulators of innate immunity, microglia, macrophages and an immune signature associated with embryonic CP, suggesting that immune function was important in CPC resistance to combination AZD1390 and radiation (Figure 5B and Table S2).

The GN^Reg-E^ also contained an expanded progenitor community like that seen in the GN^AZD1390^ and GN^IR^: 65% (*n* = 1,904/2,909) of GN^Reg-E^ progenitor community genes overlapped with those of the GN^AZD1390^ and/or GN^IR^ progenitor communities. The expanded GN^Reg-E^ progenitor community was enriched with the same cell cycle and DNA repair regulators recruited to monotherapy relapsed GNs, and Regimen-E relapsed tumors displayed increased expression of DHFR protein, and decreased expression of γH2AX protein, indicative of enhanced cell cycle and DNA repair activity, respectively (Figures S5C–S5F).

Thus, RECODR unmasked a complex pattern of gene context drift during combination AZD1390 and radiation treatment resistance that included changes induced uniquely by the combination (immune community expansion) as well as those driven by either therapy alone (progenitor community expansion).

The expansion of the immune community in Regimen-E relapsed tumors was surprising since all scRNA-seq profiles were derived from YFP^+^/RFP^+^ tumor cells that we stringently isolated by FACS (Figure S2). We reasoned that this could have resulted from increased numbers of CPC cells displaying myeloid mimicry.^39^ Indeed, the immune communities of all GNs were enriched for a myeloid mimicry signature previously described in glioma (Table S1). Alternatively, it remained possible the GN^Reg-E^ immune community originated from inadequately separated brain resident microglia and/or tumor associated macrophages.

We took two approaches to distinguish these possibilities. First, we conducted extensive multiplex profiling of cell surface antigens–including TREM2^40–42^ that marks microglia and macrophages–together with chromosome 15 copy number fluorescence *in situ* hybridization. This identified a subpopulation of high-TREM2^+^/GFP^+^/Chromosome 15-gained *bona fide* malignant cells specifically in Regimen-E relapsed CPCs that were readily distinguishable from GFP^-^/TREM2^+^/CD45^+^/ CD68^+^/P2Y12^+^/CD49d^+^ tumour-associated macrophages and GFP^-^/TREM2^+^/CD45+/CD68^+^/P2Y12^+^/CD49d^-^ microglia (Figures 5C and 5D; Figures S5I–S5K). Second, we used Visium HD spatial transcriptomics to identify cells in control and Regimen-E relapsed CPCs with abnormal karyotypes (using inferred chromosome copy number variance including gain of chromosome 15; STAR Methods) that expressed the immune, progenitor and/or mesenchymal communities. Immune community expression–as well as expression of progenitor and mesenchymal communities–was significantly more enriched in tumor cells than those with inferred diploid genomes (Figures S6A and S6B), and immune community gene expression was more enriched in Regimen-E relapsed than control CPC cells (Figures 5E, S6C, and S6D). Notably, Visium HD detected concurrent expression of progenitor, immune and mesenchymal communities within the same spatial bins, suggesting these are co-expressed in the same, rather than different, CPC cells.

**Figure 6:**
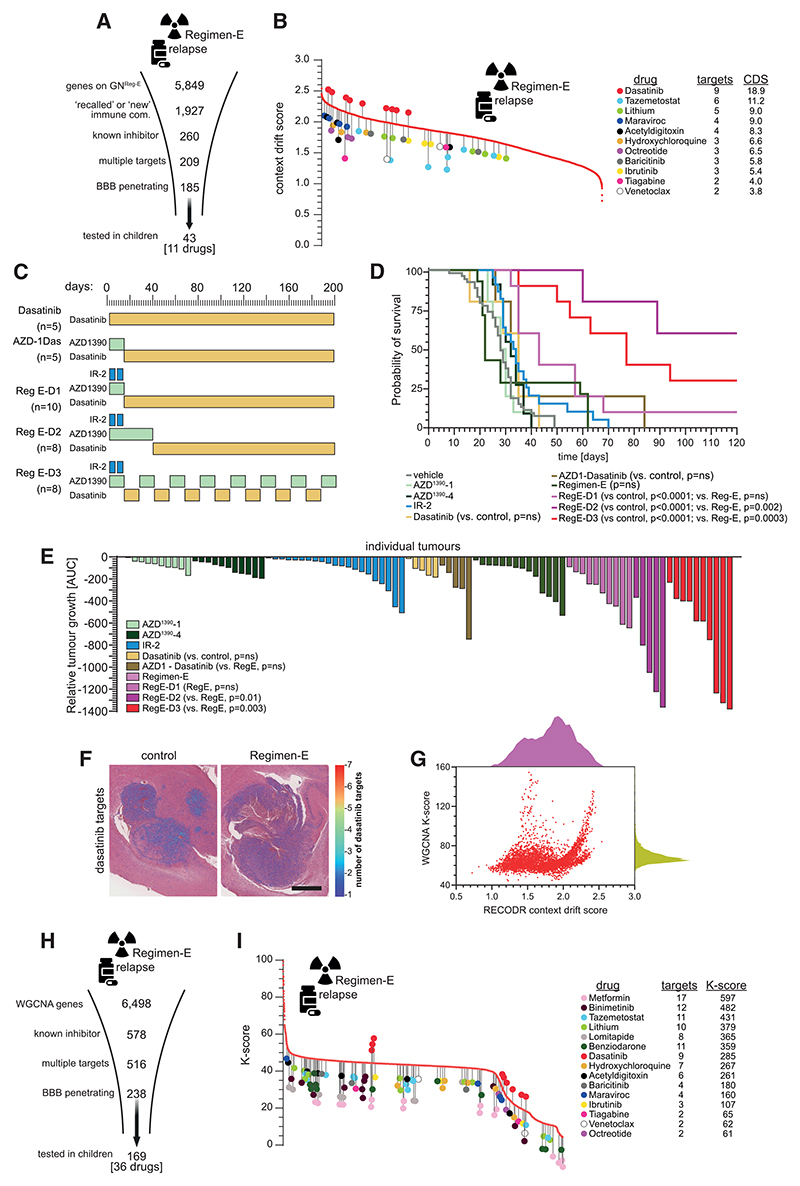
RECODR enables effective combination treatment design (A) Triage of candidate gene targets to mitigate Regimen-E resistance (BBB = blood brain barrier). (B) Ranked context drift scores of all genes on the GN^Reg-E^. Lollipops mark target genes of the indicated inhibitors. Number of targets and sum of context drift scores (CDS) are shown right. (C) Preclinical trial designs to mitigate Regimen-E resistance as well as dasatinib monotherapy and AZD1390-Dasatinib combination (n = number of mice enrolled in each arm). (D) Survival curves for the corresponding preclinical treatment protocols in (C). Monotherapy treatments are shown for comparison (*p* values report the Log-Rank statistic relative to control treatment). (E) Waterfall plots reporting growth suppression of individual tumors over time during the indicated treatment. Bars report the total area under the curve of tumor growth over time measured by weekly tumor bioluminescence relative to control treated growth (*p* values record significant difference in growth relative to controls by Mann– Whitney test). (F) Expression of dasatinib targets detected by Visium HD spatial transcriptomics in control and Regimen-E relapsed CPC (scale bar = 1mm). (G) Comparison of distributions of WGCNA K-scores and RECODR context drift scores in Regimen-E relapsed tumors. (H) Triage of candidate gene targets detected by WGCNA to mitigate Regimen-E resistance (BBB = blood brain barrier). (I) Ranked WGCNA K-scores of all genes on the GN^Reg-E^. Lollipops mark target genes of the indicated inhibitors. Number of targets and sum of WGCNA K scores are shown right.

To see if the immune community included targets to mitigate combination AZD1390 and radiation resistance, we triaged the 5,849 genes on the GN^Reg-E^ for those: (1) “recalled” or “new” to the GN^Reg-E^ immune community; (2) with clinically proven inhibitors known to cross the blood-brain barrier; (3) with inhibitors previously tested in children; and (4) with inhibitors that targeted multiple immune community genes since we reasoned these may disrupt treatment resistance the most (Figure 6A and Table S2). The multi-kinase inhibitor dasatinib ranked top among 11 drugs triaged in this manner, both in terms of the number of target genes present in the Regimen-E relapsed immune community (*Lyn, Syk, Hck, Vav1, Irak2, Fgr, Sla, Btk*, and *Nck1*) and their summated context drift scores (Figure 6B and Table S2). In stark contrast, few dasatinib targets with relatively low context drift scores were located on the GN^CPC^ (*Grb2*), GN^AZD1390^ (*Irak2, Fer, Grb2*) or GN^IR^ (*Nck1, Grb2*; Table S2). Thus, we hypothesized that dasatinib would mitigate combination AZD1390 and radiation therapy resistance but be ineffective as a primary treatment or at preventing monotherapy resistance.

To test these predictions, we randomized 36 mice with CPC among five different treatment arms: dasatinib alone; dasatinib following AZD1390 monotherapy; or dasatinib in the context of limited (Regimen E-D1), extensive (Regimen E-D2) or intermittent (Regimen E-D3) outgrowth of AZD1390 and radiation combination resistant tumors (Figure 6C). In keeping with RECODR’s predictions, neither dasatinib alone, nor AZD1390 followed by dasatinib proved effective treatments of CPC, and Regimen E-D1 was no more effective than Regimen-E alone (Figures 6D and 6E). Conversely, Regimens E-D2 and E-D3 markedly and significantly suppressed tumor growth and increased mouse survival relative to Regimen-E: 60% of mice receiving E-D2 achieved long-term survival with no evidence of relapse. Thus, RECODR accurately predicted the context-specific value of dasatinib therapy in the treatment of CPC, allowing the rationale design of a highly effective combination treatment protocol for this rare cancer.

In benchmarking studies, *Diffcoexp* detected only low levels of co-differential expression of a single dasatinib target (*Grb2*) among CPCs receiving different treatments, and no dasatinib targets were detected as differentially expressed among CPCs, regardless of treatment status (Figure 6F and Table S4). WGCNA K-scores had limited power to discriminate potential target genes in Regimen-E relapsed CPCs, and WGCNA K-scores failed to prioritize dasatinib as a useful treatment to mitigate Regimen-E relapse (Figures 6G–6I).

### RECODR predictions for clinical trials

**Figure 7:**
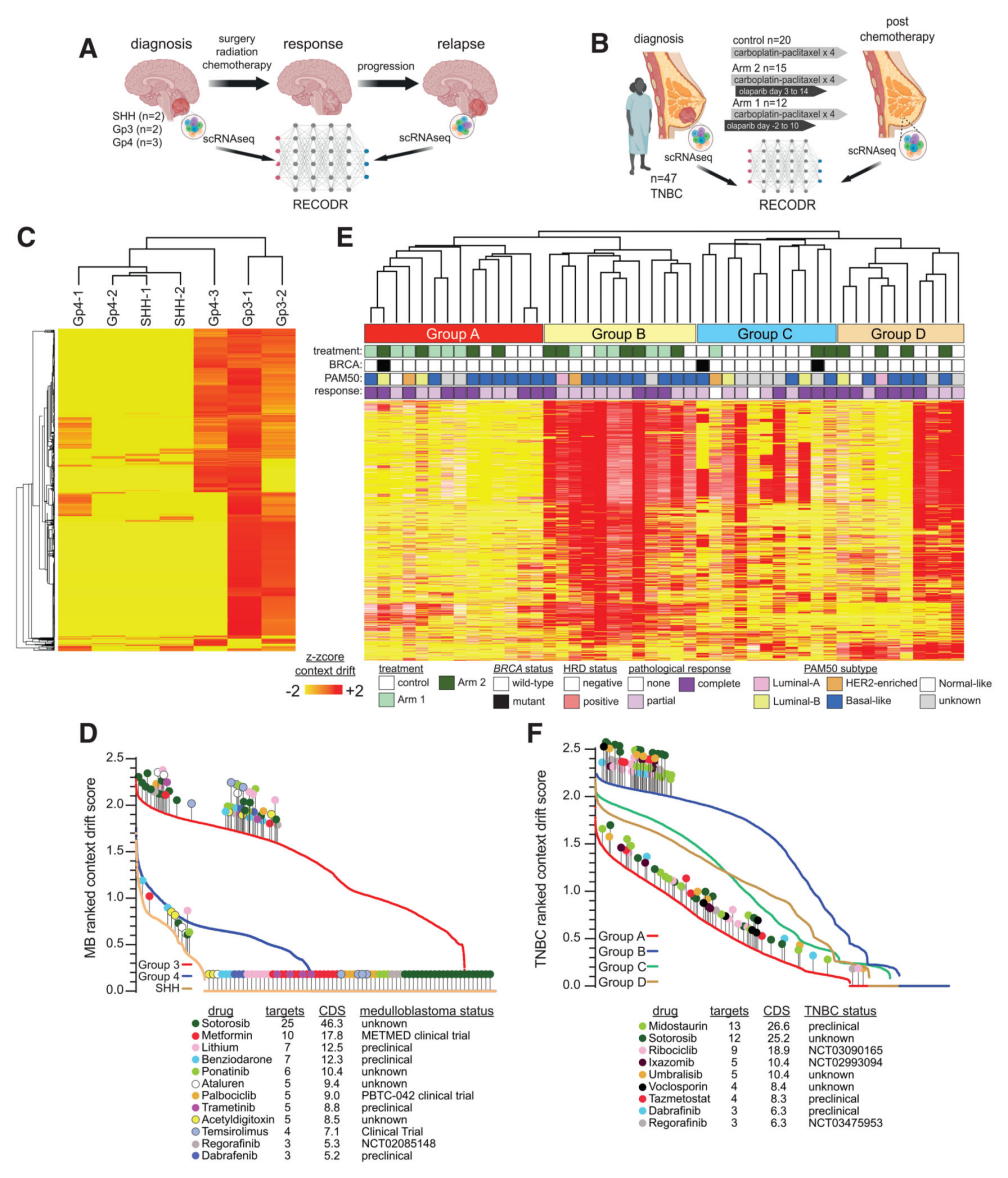
RECODR analysis of medulloblastoma and triple negative breast cancer Cartoons depicting the approach taken to analyze medulloblastoma (A) and triple negative breast cancer (TNBC, B) derived sc/snRNA-seq data by RECODR (n = number of patients with paired samples). (C) Unsupervised hierarchical cluster analysis of 4,500 genes with the most variable context drift scores in patients with medulloblastoma. Subgroup status and case number are shown. (D) Ranked context drift scores generated by RECODR of all genes in the indicated medullo-blastoma subtypes. Lollipops mark target genes of the indicated inhibitors emerging from triage of target genes. Number of targets, sum of context drift scores (CDS) and the status of the inhibitor are shown below. (E) Unsupervised hierarchical cluster analysis of 4,500 genes with the most variable context drift scores in patients with TNBC. Subgroups A–D detected by cluster analysis are shown with their corresponding treatment type from (B), *BRCA* mutational status, and neo-adjuvant treatment response. (F) Ranked context drift scores generated by RECODR of all genes in TNBCs. Lollipops mark target genes of the indicated inhibitors emerging from triage of target genes. Number of targets, sum of context drift scores (CDS) and the status of the inhibitor are shown below.

As a first step to test if RECODR could be applied to improve the treatment of patients, we generated GNs and context drift scores from pre- and post-treatment human cancer sc- or single nuclear (sn)-RNA-seq profiles (Figures 7A and 7B).

First, we analyzed paired, tumor scRNA-seq profiles generated from Sonic Hedgehog (SHH, *n* = 2), Group 3 (*n* = 2) or Group 4 (*n* = 3, including one metastatic tumor) medulloblastomas at diagnosis and following relapse after combination surgery/radiation/chemotherapy. It is well established that Group 3 and metastatic medulloblastomas are the most treatment resistant forms of the disease, but the mechanism(s) driving this resistance is not known since subtype-specific gene signatures are generally stable between diagnosis and relapse.^43,44^ Remarkably, the 4,500 genes displaying the greatest context drift between diagnosis and relapse did so within the transcriptomes of both Group-3 and a metastatic Group-4 medulloblastomas: these same genes displayed little context drift in SHH- and non-metastatic Group-4 tumors (Figure 7C and Table S6). Importantly, only 1% (*n* = 482/ 4,500) of genes with the highest context drift scores overlapped with those with the most variable levels of expression that clustered tumors by subtype (Figures S7A and S7B). Thus, RECODR context drift scores provide information that is distinct from that of gene expression level. Triaging genes with the highest context drift score in Group-3 tumors for those with inhibitors previously tested in children and that cross the blood brain barrier, identified 12 therapies that we predict might mitigate Group-3 treatment resistance (Figure 7D). Eight of these drugs are already in clinical (metformin, palbociclib, temsirolimus, and regorafenib) or preclinical (lithium, benziodarone, trametinib, and dabrafenib) development for medulloblastoma. Our data strongly suggest that these drugs are most likely to mitigate treatment resistance of Group-3 medulloblastoma and may well be judged “inactive” if tested in other tumor subtypes.

To extend the use of RECODR beyond childhood brain tumors, and to test if gene context drift can be detected immediately following treatment, rather than after relapse, we analyzed snRNA-seq profiles of TNBC generated from 47 women before, and immediately following, neoadjuvant chemotherapy as part of the PARTNER clinical trial^45,46^ (Figure 7B). TNBC is the most treatment-resistant type of breast cancer, but how these tumors fail therapy is unclear. Unsupervised hierarchical clustering of TNBCs using 4,500 genes with the most varying context drift scores identified four discrete subgroups (A to D; Figures 7E and 7F; Table S6). Although neither *BRCA* mutational status, PAM50 type, treatment arm nor treatment response varied significantly among these subgroups, Group B included 12 tumors that displayed particularly high gene context drift scores. Like medulloblastoma, TNBC subgroups identified by hierarchical clustering of context drift scores differed from those identified by gene expression level (Figures S7C and S7D). Thus, we propose that Group B represents a previously unknown subgroup of TNBC with an intrinsic capacity for marked transcriptome evolution. RECODR predicted nine treatments that might be added to Group B neoadjuvant therapy should these tumors ultimately relapse (Figure 7F). Six of these drugs are already in active clinical (ribociclib, ixazomib, and regorafenib) or preclinical (midostaurin, tazemetostat, and dabrafenib) development for TNBC.

## Discussion

Here, we introduce RECODR: a computational pipeline that combines co-expression GNs with a graph-embedding approach to measure how genes change their co-expression context within cancer transcriptomes during tumorigenesis and treatment resistance. RECODR is based on the idea that changes in gene context during therapy, rather than expression level alone, reveal important information about the evolution of tumor transcriptomes and treatment resistance mechanisms. Through a series of preclinical trials in a mouse model of CPC, we show how RECODR can detect changes in gene context that occur in response to single treatments and unmask complex changes in gene context that occur when these therapies are combined. We further show that genes undergoing the greatest context drift likely contribute the most to disease resistance and are therefore effective drug targets to mitigate treatment failure. By extending the use of RECODR to human medulloblastoma and TNBC, we provide evidence that our approach could enhance clinical trial design by identifying tumors with particularly high levels of gene context drift that might be at risk of treatment failure, and by pinpointing potential treatments to mitigate resistance. Thus RECODR, and the underpinning principle of gene context drift, provide a way to conceptualize, chart, and prevent cancer treatment resistance, particularly among cancers in which context-specific gene interactions determine the malignant phenotype.^4,5^

No current computational approaches to discover cancer drug targets are based on the concept of gene context drift.^15–19^ By deploying this approach, RECODR has the advantage of “learning the meaning” of the context of each gene within the actual tissue of interest, rather than assigning meaning to gene expression through comparison to pathways and interactions previously defined in other contexts. RECODR thereby enables the operator to rank the potential importance of genes in tumorigenesis and treatment resistance without the need for prior knowledge. Indeed, in our benchmarking studies RECODR out-performed existing conventional and advanced computational approaches–including WGCNA, diffcoexp and scDRUG– underscoring its potential added value.

Our work also places further focus on the plasticity of cancer cell transcriptomes as a means to resist cancer treatment. At least two mechanisms can bring about changes in cancer cell genotype/phenotype during treatment. It is well established that rare subpopulations of treatment-resistant cancer cells can outgrow more sensitive cells during therapy.^5,47^ But cancer cells also exhibit transcriptome plasticity, enabling individual cells to adapt their gene expression in response to microenvironmental stress, promoting cell survival. For example, the capacity of cancer cells to reacquire embryonic stem cell-like states in response to chemotherapy is well described.^5,36^ But whether these embryonic programs are reactivated in malignant stem cells–the progeny of which alter the balance of cell types in the cancer–or if gene expression is altered directly in more differentiated cancer cells, remains to be determined.^36^ RECODR showed that one-half of all significantly co-expressed genes in combination AZD1390 and radiation resistant CPCs were “recalled” from the embryonic CP to communities of genes associated with progenitor, immune or mesenchymal functions. Furthermore, the GFP^+^/RFP^+^ cells that populate our CPC mouse model included progenitor, immune and mesenchymal-like cells that shared the same chromosome copy number variations, strongly supporting the notion that these cells share a common, clonal origin. Since lineage tracing of CP has revealed a common origin for ciliated epithelial and neuronal cells,^25^ then we predict this plasticity is inherited by daughter tumors. Notably, our spatial transcriptomic studies identified co-expression of progenitor, immune and mesenchymal community genes in the same cells rather than labeling distinct tumor cells. These data are compatible with extreme plasticity in CPC transcriptomes, and with topic modeling of embryonic CP scRNA-seq profiles in which considerable temporal and topographical plasticity was seen in gene co-expression patterns across various normal CP cell types.^25^ Future work will be required to further characterize this plasticity that likely underpins the capacity of CPC cells to adapt to, and ultimately resist, treatment.

The increased number of TREM2^+^ myeloid-like tumor cells following Regimen-E combination treatment resistance was unexpected. Non-malignant, immunosuppressive myeloid cells are established contributors to tumor progression and are viewed as potential cancer treatment targets.^40,48^ But cancer cells can also adopt myeloid-like states to create an immunosuppressing tumor microenvironment.^39^ It is noteworthy that SYK —a dasatinib target that RECODR predicted could be targeted to mitigate combination treatment resistance— is central to TREM2 signaling.^42^ TREM2 is known to signal via SYK to promote myeloid cell survival in the brain and can be targeted to remodel the land-scape of tumor-infiltrating macrophages.^49^ Thus, dasatinib may target a TREM2-SYK signaling axis to prevent Regimen-E treatment failure. Importantly, since CPC cells co-expressed progenitor and immune communities, then the success of AZD1390, radiation and dasatinib combination may stem from their successful disruption of both co-expression communities and their associated functions in cancer cells. Review of combination AZD1390 and radiation clinical trials in glioblastoma and CPC will be important to determine if similar treatment resistance and mitigation mechanisms operate in these diseases.

Finally, efforts to circumvent cancer treatment resistance in the clinic have focused on combining treatments that proved effective as single agents in the hope they will evoke non-overlapping patterns of efficacy and resistance.^6–8^ Designing more rational treatment combinations has not been possible because existing approaches do not adequately predict the complex biology that drives treatment resistance; therefore, treatment resistance remains the leading cause of cancer-related death. Our studies of medulloblastoma and TNBC, together with our preclinical CPC work, suggest that RECODR could prove useful in the prospective and real-time design of effective combination cancer treatments in the clinic.

### Limitations of the study

While RECODR accurately identified ATM, PARP1, and proteins inhibited by dasatinib as useful treatment targets in CPC, testing all RECODR-predicted target genes relative to their gene context drift score is limited by preclinical experimental capacity and drug availability. We are seeking to overcome this challenge using large-scale CRISPR screens that delete 10s–100s of RECODR-predicted genes *in vivo*. Preclinical studies of other cancer types will also be required to determine how often gene context drift occurs across cancer types, and therefore how generalizable RECODR is likely to be as a cancer treatment prediction tool. While our analysis of human medulloblastoma and TNBC scRNA-seq profiles identified patients displaying high levels of gene context drift who we predict are at high-risk of relapse, further follow up these patients, and those with other cancers, it will be required to determine how accurately gene context drift predicts risk-of-relapse and mitigating treatments in the clinic. Finally, although each of the variables that make up the gene context drift score can vary independently, future studies may identify elements of the score that are consistently less informative: these may ultimately be removed from RECODR, increasing computational efficiency.

### Resource Availability

#### Lead contact

Further information and requests for resources and reagents should be directed to and will be fulfilled by the Lead Contact, Professor Richard James Gil-bertson: (Richard.Gilbertson@cruk.cam.ac.uk)

#### Materials availability

No new materials were generated as part of this study.

## Star★Methods

### Key Resources Table

**Table:** 

REAGENT or RESOURCE	SOURCE	IDENTIFIER
**Antibodies [clone where available]**		
Anti-TREM2 antibody [EPR26210-1]	Abcam	Cat#ab305103; RRID: AB_3086793
Anti-CD68 antibody [KP1]	Abcam	Cat#ab955; RRID: AB_307338
InVivoMAb anti-mouse/human VLA-4 (CD49d)	BioXCell	Cat#BE0071
Alexa Fluor® 647 anti-mouse CD45 Antibody	Biolegend	Cat#160304
Mouse monoclonal turboGFP antibody	Origene	Cat#TA150041; RRID: AB_2622256
Anti-Hck (phospho Y410) antibody	Abcam	Cat#ab61055; RRID: AB_942255
anti-Lyn antibody	Abcam	Cat#ab137338; RRID: AB_3099739
anti-Dihydrofolate reductase (DHFR) antibody	Abcam	Cat#ab152159; RRID: AB_3099740
**Culture reagents, chemicals/drugs and probes**		
Neurobasal media	ThermoFisher Scientific	Cat#21103-049
L-Glutamine	Gibco	Cat#25030-081
N2	Gibco	Cat#17502-048
B27	Gibco	Cat#17504044
Penicillin/Streptomycin	Gibco	Cat#15140-122
bFGF	Miltenyi Biotech	Cat#130-093-243
hrEGF	Miltenyi Biotech	Cat#130-097-751
BSA 7.5%	Sigma-Aldrich	Cat#A8412
FBS (heat inactivated)	Gibco	Cat#A5256801
StemPro Accutase	Gibco	Cat#A1110501
KaryoMax Colcemid	Invitrogen	Cat#15212012
Papain from papaya latex	Sigma-Aldrich	Cat#P4762
DNAseI	VWR	Cat#A3778
HBSS, no calcium, no magnesium, no phenol red	ThermoFisher Scientific	Cat#14175095
DAPI	Sigma-Aldrich	Cat#D9542
D-Luciferin	Perkin-Elmer	Cat#122799
Matrigel	Corning	Cat#354230
DMEM (1X)	Gibco	Cat#11965092
methanesulfonic acid (MSA)	Sigma-Aldrich	Cat#471356
Tween 20	Sigma-Aldrich	Cat#P1379
Tween 80	Sigma-Aldrich	Cat#P1754
Dasatinib	MedChemExpress	Cat#HY-10181
AZD1390	AstraZeneca	NA
AZD9574	AstraZeneca	NA
fish skin gelatin	Sigma-Aldrich	Cat#G7041
True Black Reagent	Biotium	Cat#23007
XMP 15 Mouse Chromosome painting probe	Metasystems	Cat#D-1415-050-OR
Fixogum rubber cement	VWR	Cat#ICNA11FIXO0125
Prolong Diamond Antifade Mountant	ThermoFisher Scientific	Cat#36961
Xylene	ThermoFisher Scientific	Cat#X/0200/17
Hematoxylin	Sigma-Aldrich	Cat#51275
Bluing buffer	Epredia	Cat#10381775
Alcoholic Eosin	Abcam	Cat#ab246824
Hydrochloric acid	ThermoFisher Scientific	Cat#J/4320/15
Potassium Hydroxide	ThermoFisher Scientific	Cat#P/5640/53
SPRIselect beads	Beckman Coulter	Cat#B23318
Mouse BD Fc Blocking solution	BD Biosciences	Cat#553141
**Critical commercial assays**		
Tissue Pretreatment Kit	OGT	Cat#LPS100
**Deposited data**		
Raw single cell RNA-sequencing data	Current manuscript	GEO: GSE252995
**Experimental models: Organisms/strains**		
mCPC model	Tong et al.^23^	Tumors generated by *in vivo* electroporation and cells maintained as allografts. Available on request
**Software and algorithms**		
R	https://www.r-project.org/	NA
Seurat	https://satijalab.org/seurat/	NA
CONICSmat	https://github.com/Neurosurgery-Brain-Tumor-Center-DiazLab/CONICS	NA
Python	https://www.python.org/	NA
Scanpy	https://scanpy.readthedocs.io/en/stable/	NA
spatialdata-io	https://spatialdata.scverse.org/projects/io/en/latest/	NA
infercnvpy	https://github.com/icbi-lab/infercnvpy	NA
NetworkX	https://networkx.org/	NA
python-louvain	https://github.com/taynaud/python-louvain	NA
Node2Vec	https://github.com/eliorc/node2vec	NA
Gensim	https://github.com/piskvorky/gensim	NA
Procrustes alignment	https://gist.github.com/zhicongchen/9e23d5c3f1e5b1293b16133485cd17d8	NA
Gephi	https://gephi.org/	NA
Cell Ranger	https://www.10xgenomics.com/support/software/cell-ranger	NA
Space Ranger	https://www.10xgenomics.com/support/software/space-ranger/latest	NA
HALO(R)	https://indicalab.com/halo/	NA

### Method Details

#### Cell culture and tissue analysis

##### Fluorescence-activated cell sorting (FACS)

Tumors were freshly isolated from mice by micro-dissecting and dissociation for 1 h at 37°C in enzymatic dissociation solution containing 20U/ml of papain (Sigma-Aldrich, P4762) and 100μg/ml of DNAseI (VWR, A3778) in high glucose DMEM (Gibco, 11965092) with 2mM L-Glutamine, 5% penicillin/streptomycin, and 10% FBS (Gibco, A5256801). Following dissociation at 37°C, cells were triturated 5–6 times before being passed through a 40μm filter. The original tube containing cells was washed with 5mL of HBSS (ThermoFisher Scientific, 14175095) which was then passed through the same 40μm filter. Cells were then centrifuged for 5 min at 4°C and 300g. Following centrifugation, the supernatant was discarded, and the cell pellet was re-suspended in 10mL HBSS before being passed through a second 40μm filter. The cell suspension was then centrifuged for 5 min at 4°C and 300g. Following centrifugation, cells were re-suspended and transferred to a FACS tube. Dual RFP and YFP cells were flow sorted using a BD Aria II (BD Biosciences) with the following gating strategy, forward and side scatter, singlets and dual RFP (561nm-585/29) and YFP (488nm-530/40) positive cells. Dead cells were excluded with DAPI, UV (355nm-450/50).

##### Cell culture

CPC cells were maintained in cell culture at 37°C with 5% CO_2_ in neurobasal media (ThermoFisher scientific, 21103-049) supplemented with N2 (Gibco, 17502-048), serum-free B27 (Gibco, 17504044), L-Glutamine (Gibco, 25030-081) and penicillin/streptomycin (Gibco, 15140-122), 100μg/ml bFGF (Miltenyi Biotech, 130-093-243), 100μg/ml hrEGF (Miltenyi Biotech, 130-097-751) and 7.5% BSA (Sigma-Aldrich, A8412). Cells were grown until neurospheres formed. For cell passaging, neurospheres were centrifuged at 50g before being passed through a 40μm filter. Flasks were then rinsed with 5mL PBS and passed through the same filter. 2mL of StemPro Accutase (Gibco, A1110501) was then added and cells dissociated for 15 min at 37°C. Neurospheres were then triturated and passed through a 40μm filter into 20mL of media. Cells were then centrifuged at 118g for 5 min before being re-suspended in complete media at a 1:2 dilution.

##### Chromosome painting

Cells were plated in 6-well plates (1.5million cells/well) and incubated for 24 h. Colcemid (Invitrogen, 15212012) was added to arrest cells in metaphase and incubated for 6 h. After 6 h, cells were triturated to dissociate clumps, collected in 1mL PBS and centrifuged at 400g for 8 min at room temperature. 10mL of hypotonic solution (0.0075M KCl in dH2O) was added, and cells were incubated for 12 min. After 12 min, 1mL of fixative (3:1 methanol: acetic acid) solution was added, and cells were centrifuged at 400g for 8 min at room temperature. Following centrifugation, the supernatant was removed, and 5mL of fixative solution was added and centrifuged at 400g for 8 min at room temperature. This washing step was repeated three times followed by dropping onto slides. The slides were allowed to dry for 3 h. Metaphase was then confirmed by staining with DAPI (Sigma-Aldrich, D9542) followed by two washes with PBS and imaging by confocal microscopy.

For multiplex fluorescence *in situ*, hybridisation (M-FISH), 10μL of 24 colors human M-FISH paint probe mix generated at the Wellcome Trust Sanger Institute as previously described^50^ was denatured at 65°C for 10 min and then applied to the denatured slides. Slide denaturation was performed by immersing slides into alkaline denaturation solution (0.5M NaOH and 1M NaCl) for 40 s before being rinsed with 1M Tris-HCl at pH 7.4 for 3 min followed by 1X PBS for 3 min and then dehydrated with 70%, 90% and then 100% ethanol. For hybridisation, slides were incubated for 10 min at 37°C for 40–44 h. After incubation, slides were washed with 0.5X saline sodium citrate (SSC) for 5 min at 75°C, then rinsed with 2X SSC +0.05% Tween 20 for 5 min and washed with 1X PBS for 2 min at room temperature. Slides were then mounted with SlowFade Diamond Antifade Mountant containing DAPI. Images were taken on a Zeiss AxioImager D1 fluorescent microscope. M-FISH images were captured with the SmartCapture software (Digital Scientific UK) and processed with SmartType Karyotypes software (Digital Scientific UK).

##### Immunofluorescence staining

Formalin fixed paraffin embedded tissue sections were first deparaffinized and treated using a standard antigen unmasking step in 10 mM Tris/EDTA buffer pH 9.0. Sections were then blocked with Mouse BD Fc Blocking solution (BD Biosciences, 553141) and treated with True Black Reagent (Biotium, 23007) to quench intrinsic tissue autofluorescence.

Detection of mouse chromosome 15 was performed on FFPE sections. Pretreatments were carried out using the Tissue Pretreatment Kit (OGT, LPS100) according to manufacturer’s instructions: Incubation with Tissue Pretreatment Solution (Reagent 1 at 98°C for 10 min followed by 3 min wash in milliQ water twice, 8 min incubation with Enzyme Reagent (Reagent 2) at 37°C followed by 3 min wash in milliQ water twice). Slides were dehydrated through graded ethanol. Ready-to-use XMP 15 Mouse Chromosome painting probe (Metasystems, D-1415-050-OR) was applied to each slide and coverslips were sealed with Fixogum rubber cement (VWR, ICNA11FIXO0125). Slides were denatured for 5 min at 75°C before hybridisation overnight in a humid chamber at 37°C. Rubber cement was carefully peeled off and coverslips were removed by soaking in 2X SSC +0.05% Tween 20 (Sigma-Aldrich, P1379) followed by a wash in 0.4X SSC buffer at 72°C for 2 min and 2X SSC +0.05% Tween 20 for 30 s. Slides were incubated with DAPI for 5 min at room temperature in the dark. Slides were washed for 5 min each in 3 changes of PBS prior to mounting with Prolong Diamond Antifade Mountant (ThermoFisher Scientific, 36961).

The sections were then treated with a 1-min additional heat mediated antigen retrieval step in Tris/EDTA buffer. The sections were then immunoreacted for 1 hour at RT using 1μg/ml cocktail mixture of immunocompatible antibody panels (see key resources table for antibody sources). This step was followed by washing off excess primary antibodies in PBS supplemented with 1 mg/mL fish skin gelatin (Sigma-Aldrich, G7041) and staining the sections using a 1μg/ml cocktail mixture of the appropriately cross-adsorbed secondary antibodies (purchased from either ThermoFisher Scientific, Jackson ImmunoResearch or Li-Cor Biosciences) conjugated to one of the following spectrally compatible fluorophores: DyLight 405, Alexa Fluor 488, Alexa Fluor 546, Alexa Fluor 594, Alexa Fluor 647, Cy5.5 or IRDye 800CW. After washing off excess secondary antibodies, sections were counterstained using DAPI to visualize cell nuclei. Coverslips were then placed on slides using Prolong Diamond Antifade Mountant and imaged using the Vectra Polaris (described in ‘image acquisition‘ below).

##### Image acquisition

All fluorescently labeled slides were scanned on the Vectra Polaris at 40X magnification using appropriate exposure times. Whole-slide images were scanned with 7-color whole-slide unmixing filters (DAPI + Opal 570/690, Opal 480/620/780 and Opal 520). Library slides were generated from representative tissue sections to allow for accurate unmixing of the multiplexed samples, including a slide stained for each single fluorophore, a DAPI only slide and an autofluorescence slide wherein no antibody, or DAPI was applied. The unmixing performance of this tissue-specific spectral library was compared to that of the synthetic Opal library available in inform. Resultant image tiles were then stitched together within HALO(R) (Indica Labs) to produce a whole-slide multichannel, pyramidal OME-TIFF image for downstream imaging analysis.

##### shRNA screen

shRNAs were generated and used in knock-down studies as described previously.^51^ Briefly, shRNAs targeting each candidate DNA repair enzyme (or scrambled) were designed using the shRNA sequence prediction algorithm from Dharmacon/Thermo Scientific. shRNAs were cloned into the pFUGWH1-CFP vector and transformed into bacteria as one ligation product. The transformed bacteria were then spread on an LB/AMP plate and individual colonies were screened for unique shRNA constructs by sequencing. All constructs were sequence verified. DNA was fugene (Roche) transfected into 293FT cells (Invitrogen) along with lentiviral packaging plasmids for viral production. Virus titer was determined by cyan fluorescence protein (CFP) expression and transduced into CPC, e3 Cancer Cell *43*, 1608–1621.e1–e9, September 8, 2025 RTBDN-ependymoma or mouse neural stem cells. Cells were then plated as single cells and colony formation determined after 72–120 h.

#### Single cell and spatial transcriptomics

##### Single cell RNA-sequencing (scRNAseq)

Sorted cells derived from untreated (*n* = 2), AZD1390 relapsed (*n* = 3), radiation relapsed (*n* = 3), or Regimen-E relapsed (*n* = 6) CPCs were submitted for 10X sequencing and libraries were prepared using the Chromium Single Cell 3^′^ Reagents kit v.3. Briefly, samples were resuspended in PBS with 0.04% BSA and loaded onto the Chromium microfluidic chip (10X Genomics) to generate single-cell bead emulsions with individual 10X barcodes. RNA from barcoded cells was reverse transcribed in a C1000 Touch Thermal cycler (Bio-Rad) and libraries were prepared according to manufacturer’s protocols (12 cycles were used for cDNA amplification). cDNA quantity and quality were measured using an Agilent TapeStation 4200 (High Sensitivity 5000 ScreenTape). About 375ng of material was used for library preparation and library quality was assessed with a TapeStation 4200 (High Sensitivity D1000 ScreenTape). Libraries were normalized to equal molar concentrations (10nM) and pooled. Sample pools were sequenced on a NovaSeq 6000 to the following parameters: 28bp, read 1; 8bp, i7 index and 91bp, read 2, aiming for 20000 reads per cell. RNA reads were processed by Cell Ranger (10x genomics) and aligned to the mouse (mm10) genome. The filtered gene matrices were then used as the input for downstream analysis pipelines.

##### Single cell/nucleus RNA-sequencing analysis

scRNAseq was analyzed in R using Seurat. First, for each condition, individual samples were read into R using the *Read10X* function followed by merging samples from the same condition using the *merge* function. The *PercentageFeatureSet* function was then used to calculate the percentage of counts derived from mitochondrial genes. The data was filtered to include cells with more than 200 genes, less than 3000 genes and less than 20% mitochondrial content per cell. Next, cells were log normalized using the *NormalizeData* function. The *FindVariableFeatures* and *ScaleData* functions were then run to find the top 2000 most variable features and scale the dataset (with total number of RNA counts and percent mitochondria regressed out). Downstream clustering was then carried out by applying the *RunPCA, FindNeighbors, FindClusters, RunUMAP* and *RunTSNE* functions using default parameters. Differential expression between different cell types was carried out with the Seurat pipeline run on merged control and relapse setting datasets and then using the Wilcoxon Rank-Sum test with the *FindMarkers* function with default parameters for Seurat version 4. Normal mouse choroid-plexus single-cell datasets (GSE168704) were processed using the standard Seurat workflow. Human single nucleus RNA-sequencing (snRNAseq) of human CPC (GSE264154) were processed using the standard Seurat workflow.

##### Visium HD

Formalin fixed, paraffin-embedded (FFPE) sections of each tumor tissue were cut to a thickness of 5μm with the 6.5 mm × 6.5 mm region of interest (ROI) placed centrally on the slide and then deparaffinized. Briefly, tissue sections were incubated at 60°C, cooled and then incubated in xylene (ThermoFisher scientific, X/0200/17) twice (two separate jars of xylene) for 10 min each. Next, slides were incubated in 100% ethanol twice (two separate jars of 100% ethanol) for 3 min each. Slides were then incubated in 96% ethanol twice (two separate jars of 96% ethanol) for 3 min each before finally being incubated in 70% ethanol twice (two separate jars of 70% ethanol) for 3 min each and then washed in water twice for 20 s each. The deparaffinized slide was incubated in hematoxylin (Sigma-Aldrich, 51275) for 1 min before hematoxylin was removed with 3 washes in water. Bluing buffer (Epredia, 10381775) was then added to the slide and incubated for 1 min before being washed in water. Next, the slide was incubated in alcoholic Eosin (Abcam, ab246824) for 1 min. Finally, the slide was washed by incubating in water for 30 s and coverslips applied over tissue sections.

Deparaffinized slides were placed in the 6.5mm Visium HD cassettes with the gasket mounted over the ROI. Tissues were destained with 0.1N hydrochloric acid (ThermoFisher scientific, J/4320/15) and incubated for 15 min at 42°C before 10X decrosslinking buffer was added to tissue sections and incubated for 30 min at 80°C. The 10X mouse whole transcriptome probes were hybridized overnight by adding the 10X FFPE hybridization buffer, nuclease free water and left- and right-hand side whole transcriptome mouse probes. Hybridization was carried out at 50°C. Probe ligation was then carried out with a master mix of nuclease free water, 10X probe ligations buffer and 10X probe ligation enzyme and incubated for 1 h at 37°C. During probe ligation, Visium HD slides were brought to room temperature and washed in 0.1X SSC. After probe ligation, tissue sections were washed in 10X post-ligation wash buffer for 10 min at 57°C (two washes of 5-min each). Visium HD slides were equilibrated by adding a master mix of nuclease free water, 10X RNase buffer and 10X RNase enzyme in the capture regions and incubated for 10 min at room temperature. Slides were then allowed to dry. Slide information was entered into the CytAssist. Tissues were re-stained with alcoholic Eosin for 1 min at room temperature before being washed with PBS. Slides were then loaded onto the CytAssist and ROIs centered within the 6.5 mm × 6.5 mm fiducial frame. The probe release mix composed of 10X RNase buffer, 10X RNase enzyme and 10X permeabilization Enzyme B was prepared and added into the spacer well of the Visium HD slide. The CytAssist was then run for 30 min at 37°C to release probes from tissue sections to 8μm bins of the Visium HD slide. After 30 min the Visium HD slide was removed and probes were extended with a master mix of 10X extension buffer and 10X extension enzyme for 1 h at 53°C (two washes of 30 min each). Probes were then eluted from the Visium HD slide with 0.08M potassium hydroxide (ThermoFisher scientific, P/5640/53) and incubated for 10 min at room temperature. Solutions from every capture area of the Visium HD slide were transferred into PCR tubes and neutralized with 1M Tris-HCl. Pre-amplification buffer composed of nuclease free water, 10X Amp-mix B and 10X TS Primer mix was added and 10-cycles of pre-amplification were carried out on a thermal cycler. The pre-amplification product was cleaned up with 1.2X SPRIselect beads (Beckman Coulter, B23318) and the 100μL eluate was taken forward for library preparation. Visium HD libraries were prepared by adding an amplification master mix composed of nuclease free water and 10X Amp mix B and samples indexed with dual index TS Set A index and then incubated in a thermal cycler. Libraries were cleaned up with 0.85X SPRIselect beads resulting in a 25μL eluate. Library quality was confirmed on the Agilent TapeStation 4200. Libraries were quantified with a qubit and then normalized to 10mM. Finally, samples were pooled and sequenced on a NovaSeqX to a target of 275 million reads per capture area to the following parameters: 43 bp, read 1; 10 bp, i7 index; 10bp, i5 index and 50 bp, read2.

##### Space ranger

Space Ranger was run on FASTQ files for each sample. Folders for each sample contained their corresponding FASTQ files, a TIFF image of the H&E and a TIFF image taken from the Visium CytAssist instrument. The Space Ranger ‘count’ pipeline was then run with the following options, –id set to the experiment name, –transcriptome set to the mm10-2020-A mouse genome reference, –probe-set set to to the ‘Visium_Mouse_Transcriptome_Probe_Set_v2.0_mm10-2020_A’ which was supplied with Space Ranger, –fastqs, –cytaimage, –image set to the experiments FASTQ files, CytAssist image and H&E image respectively, –create-bam set to False, –reorient-images set to True, –slide set to the slide ID and –area set to the slide area the H&E was assigned to on the Visium slide. All remaining options were kept at their default parameters.

##### Data analysis

The Space Ranger outputs were processed in Python. First, the Space Ranger outputs were converted to a Visium HD object using the *visium_hd* function from the spatialdata_io package with the ‘filtered_counts_file’ set to True to use the filtered feature matrix. Next, the *to_legacy_anndata* function from spatialdata_io was used to convert the Visium HD object to an anndata object for processing in Scanpy with the following arguments, ‘coordinate_system’ set to ‘downscaled_hires’, ‘table_name’ set to ‘square_008um’ to select the 8μm bins and ‘include_images’ set to True. All subsequent analysis of the Visium HD data was carried out in Scanpy on data with 8μm bins. First, all gene names were made unique using the *var_names_make_unique* function. The percentage of mitochondrial genes in each bin were then calculated using the *calculate_qc_metrics* function before bin filtering was carried out. Bins were filtered to include a minimum of 100 total counts and less than 20% mitochondrial content as well as genes that were expressed in at least 10 bins. Next, the data were normalized using the *normalize_total* and *log1p* functions. Highly variable genes were detected using the *highly_variable_genes* function before downstream clustering was carried out using the *pca, neighbors, umap* and *leiden* functions with default parameters. Enrichment of gene signatures was calculated using the *score_ genes* function with default parameters. The number of dasatinib targets was determined for each cell by counting how many targets had an expression value more than 0.

##### Inferred copy number variation analysis

*Infercnvpy*. To infer copy number variation (CNV), all Seurat objects generated in ‘Single cell RNA-sequencing analysis’ (including GSE168704) were merged using the *merge* function and the R library DropletUtils was used to generate the merged single cell dataset in the traditional Cell Ranger format. The *write10xCounts* function of DropletUtils was used to generate this format using raw counts of the Seurat object. The remainder of the analysis was carried out in Python. The output of *write10xCounts* was read in as a single cell object in Scanpy using the *read_10x_mtx* function. Next, raw counts were log normalized using the *normalize_total* and *log1p* functions from Scanpy. Then, the infercnvpy package was used to infer CNV in our single-cell dataset. Following normalization, genomic positions were added to the Scanpy dataset using the *genomic_position_from_gtf* function with the gencode vM23 primary assembly. Next, the *infercnv* function was run with normal choroid plexus cells (3V, 4V and LV single-cells from GSE168704) as the reference category and default parameters. Clustering was run with infercnvpy with the *pca, neighbors* and *leiden* functions using default parameters. Plots were generated using the *chromosome_heatmap* function.

*CONICSmat*. To infer CNV in Visium HD data, the CONICSmat^52^ package in R was used. For each slide, the raw counts from the ‘filtered_feature_bc_matrix.h5’ Space Ranger output were read using the *Read10X_h5* function in Seurat. Like the downstream analysis of the Visium HD data using Scanpy, only bins containing a minimum of 100 total counts and less than 20% mitochondrial content were used. The *normMat* function was called first on raw counts for each slide. Then, gene positions were calculated using the *get-GenePositions* with ‘ensembl_version’ set to ‘sep2019.archive.ensembl.org’ and ‘species’ set to ‘mouse’. Next the *filterMatrix* function was used with the argument ‘minCells’ set to 200. *calcNormFactors* was then run with default parameters before the *plotAll* function was used with default parameters and ‘regions’ set to a.txt file of chromosome positions for mm10. To cluster cells into malignant and non-malignant categories, a Seurat object of the CNV scores was generated. Normalization was not carried out, and *ScaleData* and *RunPCA* functions were run using default parameters. *FindNeighbors was then* run with ‘dims’ set to 1:5. Finally, *FindClusters* was run with the argument ‘resolution’ set to 0.1. Based on this clustering, bins were assigned as either malignant or non-malignant. Frequency distribution plots were then generated based on this categorization plus the enrichment of graph communities calculated in Scanpy as described in ‘visium HD, data analysis‘.

##### *In vivo* therapeutics

All animal work was carried out under the Animals (Scientific Procedures) Act 1986 in accordance with the UK Home office license (Project License PP9742216) and approved by the Cancer Research UK Cambridge Institute Animal Welfare and Ethical Review Board. Mice were housed in individually ventilated cages with wood chip bedding plus cardboard fun tunnels and chew blocks under a 12-h light/dark cycle at 21 ± 2°C and 55% ± 10% humidity. Standard diet was provided with *ad libitum* water. All mice were housed for habituation for at least 1 week before the start of the experiment. The Crl:CD1-*Foxn1nu, 086* mouse strain was used. Mice for all experiments were between 7 and 9 weeks old at the start of the experiment. Mice were orthotopically implanted with 5000 CPC cells in 5μL of matrigel (Corning, 354230) per mouse. CPC cells below passage 14 were used for tumor implants. Tumor growth was monitored by bioluminescent imaging (BLI). 3 days post implant, the tumors reached a suitable signal of 5*10^5^ p/s/cm2/sr (BLI) and animals were then randomized into control and experimental groups. Mice were continually monitored for clinical signs and tumor progression was measured by BLI. For BLI monitoring, mice were intraperitoneally (IP) injected with 15mg/ml of D-Luciferin (PerkinElmer, 122799) and placed into a housing chamber containing isoflurane. Once anesthetized, mice were placed into the IVIS spectrum and positioned into a nose-cone with continued delivery of isoflurane. BLI was then measured.

##### Drug preparation

AZD1390 (AstraZeneca) was diluted into a vehicle of water +0.1% Tween 80 (Sigma-Aldrich, P1754) at 2mg/ml stock concentration. The drug was dissolved with a magnetic stirrer at room temperature and left to continually stir until administration. Dasatinib (MedChemExpress, HY-10181) was prepared in 80mM of citric acid monohydrate at 2.5mg/ml and manually mixed, then stored at 4°C. AZD9574 (AstraZeneca) was prepared in deionized water in methanesulfonic acid (Sigma-Aldrich, 471356) at a pH of 3.0– 3.2. 0.3mg/ml of AZD9574 was diluted in the vehicle and manually mixed and stored at 4°C. All drugs were allowed to come to room temperature before administration.

##### Radiation

Mice receiving radiation were anesthetized by isoflurane in a housing chamber before being put into the Small Animal Radiotherapy Research Platform (SARRP). Mice were positioned into nose-cones in the SARRP with continued isoflurane delivery. 20Gy of targeted radiation to the implant site was given to animals with 2Gy/day in cycles of 5 days on and 2 days off.

##### Drug treatment

For mice undergoing drug treatments, drugs were delivered by oral-gavage (Instech Laboratories plastic feeding tubes, 20ga x 38mm, Linton Instrumentation, FTP-20-38) daily according to treatment schedules outlined in this paper. AZD1390 was administered at a concentration of 20mg/kg, Dasatinib was administered at a concentration of 25mg/kg and AZD9574 was administered at a concentration of 3mg/kg.

##### Tissue collection and processing

Once mice reached a humane clinical endpoint, mice were sacrificed by a rising concentration of CO_2_. Brains were harvested by immediate decapitation, posterior to the occipital bone followed by removal of the brain. Brains were fixed in 10% neutral buffered formalin for 24 h, followed by 70% EtOH for a further 72 h before being paraffin-embedded. For immunohistochemical studies, 7μm thick sagittal sections were used.

#### Resistance through context drift (recodr)

##### Graph networks and community detection

For each condition (including GSE168704 and GSE264154), log-normalized counts were taken from the Seurat objects from the ‘single cell RNA-sequencing section’ and converted to dataframes. Next, the dataframes were filtered to include only genes that were expressed in at least 5% of cells. A pairwise Pearson correlation analysis was carried out between every pair of genes using the Pandas *corr* function in Python to identify the extent of linear correlation between gene pairs. The correlation matrix was then stacked using the Pandas *stack* function to generate a source, target and correlation value edgelist. Gene pairs with a Pearson correlation coefficient of 0.1 or above were taken forward for subsequent analysis. Pearson correlations between the same gene were removed to prevent self-connections in the graph network. The NetworkX function *from_pandas_edgelist* was used to generate an undirected and unweighted graph network for each condition following the steps described above where nodes represent genes, and edges connect nodes if those genes have a Pearson correlation of 0.1 or above. Community detection was carried out by the *best_partition* function from the python-louvain library using default parameters to identify modules in our graph networks. For plotting, NetworkX graphs were saved to the Gephi format with the *write_gexf* function and then plotted using the freely available software Gephi.

##### g:Profiler analysis of communities in graph networks

g:Profiler analysis of communities was carried out using the g:Profiler Python package incorporated into a newly built function that uses each list of genes in each community of a graph and runs the default g:Profiler pipeline, with or without a custom GMT file. Graph networks were generated for Pearson correlations of 0.8 to 0.1 in increments of 0.1. Iterative community detection and gene set enrichment analyses across each graph network was performed to select the final Pearson threshold. While increasing the correlation stringency from 0.1 to 0.8 reduced substantially the number of genes in each community, it did not change the pattern of functional gene enrichment in each community. We thereby employed the lower Pearson correlation threshold of 0.1.

##### Node2Vec and Word2Vec

To leverage the use of vector-based representations of nodes on each graph we employed Node2Vec followed by the skip-gram objective of the Word2Vec architecture to learn the graph structure for each condition.

To represent the graph networks in a format that adequately represents node features, we used Node2Vec.^27^ Node2Vec carries out biased random walks (second order random walks) that sample neighborhoods around nodes. Briefly, normalized transition probabilities were calculated for every node such that the likelihood of transitioning from one node to another is determined. The walks are influenced by two hyperparameters which determine the likelihood of returning to the node the walk originated from or going outward from the current node. These hyperparameters are the return parameter *p* which we set to 1 and the ‘in-out’ parameter q which we also set to 1. 200 walks were carried out from every single node on each graph for a walk length of 80 nodes. This random walk represents the graph context and generates the input format for Word2Vec.

Word2Vec^28^ is a neural network which aims to understand the context in which words are found in text, generating rich semantic representations of words. This is achieved through static vector representations of each word in a corpus of text where vectors are optimized such that words that are found in the same context have similar vectors and words that are not found in the same context have dissimilar vectors. The resulting word vectors describe the relationship of that node and all others around that node. The similarity between vectors can be measured by metrics such as the cosine distance. The Node2Vec library used internally calls on Word2Vec from the Gensim natural language processing library. We ran the skip-gram objective with negative sampling of the Word2Vec architecture with a vector ‘dimension’ of 64, ‘window’ size of 10, ‘min_counts’ of 5, initial learning rate (‘alpha’) of 0.025 and 5 negative words (‘negative’). All models were trained for 10 epochs.

##### Alignment of Word2Vec models

Training of each Word2Vec model is inherently stochastic due to the random weight initialization step when models are created. For this reason, the vectors for the same node on a graph cannot be compared to the same node on another graph immediately after training as the vector spaces will be different. To measure how each gene changes context as a function of treatment condition, we carried out the Procrustes alignment between the embedding matrices of our Word2Vec models in a pairwise manner. The alignment is an orthogonal transformation of an embedding matrix such that a word embedding matrix for one model is aligned to a target matrix from another model. This is carried out using only common words between both models. The aim is to find the orthogonal matrix that best maps to the target embedding matrix to which the model is being aligned allowing for comparisons of the same word across models. This is achieved by minimizing the sum of square distances between vectors and is solved by the singular-value decomposition (SVD).^53^

##### Comparing vectors across models

Following Word2Vec model alignment, the cosine similarity was used as a measure of context drift for words between two models. To generate the cosine distance between two vectors, the cosine function in SciPy was used to compare the same gene in two different Word2Vec models. Cosine similarity was taken as one minus the cosine distance yielding a range of values between -1 and 1. For genes that are absent in either the baseline model or comparator model, we assigned a value of -1.1. Any genes present in the aligned model but not present in the baseline model were given a value of 1.2. Any value outside the range of -1 to 1 are indicator values and do not represent a metric as no vector arithmetic is carried out.

We performed iterative analyses to optimise the vector size (64) and ‘q’ (1) parameter. Increasing the dimensionality to 100 or biasing the walk toward more outward exploration with a ‘q’ parameter of 0.7 did not improve performance as determined by the cosine similarities of aligned vectors between conditions. A context window of 10 adequately understood the wider context around each node in each condition.

##### Gene context drift score

To identify potential drug targets in the relapse setting we generated a gene context drift scoring system based on the context drift of genes from graph-embedding analysis plus characteristics of each gene on the graph network. For each gene on a graph in the relapse condition, a cumulative score was generated using four scoring criteria. The first criteria was a neighbor score. For each gene, its edges were compared to the original CPC graph. The number of edges that were unique to the relapse setting were counted and normalized to the total number of nodes in the relapse graph. The second criteria was a graph reach score. As a proxy for how much of the graph a node could reach, we took the neighbors of a node plus the unique edges of the 2nd hop neighbors. The number of unique genes was normalized to the total number of genes in the relapse graph. The third criteria was neighborhood context drift. The cosines with the relapse graph as the baseline were taken and compared to the untreated CPC Word2Vec model. Next, the cosine dataframe was subset to include only the neighbors of the node being analyzed. Genes that were not present in the untreated CPC graph were removed from the dataframe and the mean of the remaining cosines was taken as a measure of overall context drift. one minus the mean context drift was taken to reward higher context drift of genes in the relapse setting relative to the untreated CPC graph. The fourth criteria was the gene context drift. For every gene, the cosine similarity between the vectors for that gene in the relapse graph and the untreated CPC graph were taken. One minus the cosine similarity was then taken to generate a score. Any gene that was present in the relapse graph but not the untreated CPC graph was given a score of 1. The sum of all scores was taken to generate a final score for each gene. Since each of these metrics can vary independently, gene context drift score presents a powerful, holistic approach to summarise changes in context between tissue states. The ‘neighborhood context drift score’ is essential as it measures the average context drift of all the neighbors of an ‘index gene’. This is akin to how much transcriptional remodeling is happening around each gene with respect to gene context. Similarly, the ‘index gene score’ measures how much the gene in question has changed its context relative to itself between GNs. These two scores are important to combine as they give an indication of how much context drift has taken place around a gene and are central to our hypothesis. Graph connectivity contributes by further characterising the degree to which targets are associated with gene communities.

##### Circos plot

For chromosomal translocations, ‘recipient chromosome’ and ‘donor chromosome’ tables were created with the proportion of translocations observed. The *chordDiagramFromDataFrame* function from the Circlize package in R was then used to generate the circos plot.

##### Alluvial plots

Alluvial plots were generated using the open source software RAWGraphs 2.0.^54^

##### Value scaling

Plots indicating scaled-scores were scaled using the sci-kit learn library in Python with the *minmax_scale* function. Scaling was carried out across the ‘sample’ axis in order to ensure the feature range of values was scaled across the sample of interest.

##### Benchmarking

To benchmark our approach, we tested RECODR against WGCNA,^34^ Diffcoexp (https://github.com/hidelab/diffcoexp) and scDrug which utilizes the CaDRReS-Sc model.^35^ We ran WGCNA and diffcoexp in R and scDrug using a Python implementation within the package Omicverse.^55^ WGCNA was chosen as a benchmark because it is a widely used and relatively well-established approach to study gene co-expression graph networks. WGCNA also identifies modules of correlated genes in a manner similar to the ‘communities’ identified by RECODR. Benchmarking against diffcoexp allowed assessment of whether gene context drift was superior to differential co-expression as a prediction of drug targets. Finally, scDrug was chosen as a benchmark to ensure to test if direct analysis of a pre (or post) transcriptome alone rather than comparing gene context drift between pre- and pos-treatment samples was equally effective and predicting drug targets.

##### WGCNA

Counts for genes expressed in at least 5% of cells for each CPC condition (untreated CPC, AZD1390-monotherapy, IR-monotherapy and RegE) were read into R using the *read.csv* function. Parameters were set as detailed below in accordance with the recommendations of the publishers of WGCNA and following extensive reviews of online forums published by research groups using the computational pipelines. The *pickSoftThreshold* function of WGCNA was used with the ‘networkType’ parameter set to ‘signed’. The *blockwiseModules* function in WGCNA was used for network construction with ‘deepSplit’ set to 2, ‘pamRespectDendro’ set to False, ‘minModuleSize’ set to 30, ‘maxBlockSize’ set to 4000, ‘reassignThreshold’ set to 0, ‘mergeCutHeight’ set to 0.25 and corType set to ‘pearson’. The power selected was determined by the *pickSoftThreshold* for each dataset and was assigned as an R^2^ value of above 0.8. Hub genes were assigned using the *chooseTopHubInEachModule* in WGCNA and intramodular connectivity (kTotal and kWithin) were generated using the *adjacency* function in WGCNA to generate an adjacency matrix which was then passed into the *intramodularConnectivitiy* function of WGCNA.

##### Diffcoexp

To run diffcoexp, CPC counts from individual conditions were read into R using the *read.csv* function. For all analyses, we carried out drug resistant CPCs versus untreated CPC. Dataframes were subset to include only genes that were present in both conditions (a requirement of the diffcoexp tool). These dataframes were then passed to the *diffcoexp* function with ‘r.method’ set to ‘pearson’, ‘rth’ set to 0.1, ‘qth’ set to 0.1, ‘q.method’ set to ‘BH’ and ‘r.diffth’ set to 0.1. Dataframes were saved using the *write.csv* function for downstream analysis. Scatterplots with corresponding histograms of data distribution were then generated in Python. Briefly, differential edge co-expression csv files were read into Python using the Pandas *read_csv* function. Then, the dataframes were subset to include genes that were co-expressed with a pearson correlation threshold of 0.1 or above for gene one and gene two. Next, the mean of the difference in co-expression between gene pairs was taken for every gene using the *groupby* function in Pandas. The context drift scores of drug-resistant CPCs versus untreated CPC was read using the Pandas function *read_csv*. Scores were subset to include only genes that were present in the differential edge co-expression analysis before dataframes for the mean co-expression difference and context drift scores for the corresponding genes were merged using the Pandas *join* function.

##### Omicverse (for scDrug)

The Omicverse package was used to run scDrug in Python. First, H5AD objects of CPC single cell objects were read into Python using the Scanpy function *read_h5ad*. Each scanpy object was subset to include only genes that were expressed in at least 5% of cells as per the input to RECODR. As cell clusters with cell types were annotated, we ran scDrug using the clusters previously calculated. Because CaDRReS was trained on human cancer cell lines, all genes were made uppercase. Then, the adata object was passed to the *Drug_Response* class in Omicverse to generate drug predictions. The ‘GDSC’ model was used, with the number of drugs (‘n_drugs’) set to 200.

#### Human tumor analysis

All human tumor samples were collected and analyzed in accordance with Institutional Review Board (or equivalent) approvals and clinical trial consent. Medulloblastoma samples were collected children undergoing standard of care treatment following informed consent. The PARTNER trial protocol (NCT03150576 and EudraCT: 2015-002811-13) was approved by Northwest—Haydock Research Ethics Committee (ref. 15/NW/0926) and the trial was performed in accordance with the Declaration of Helsinki and the European Clinical Trials Directives 2001/20/EC (see Abraham et al.^45,46^).

##### Triple negative breast cancer

We analyzed snRNAseq data from 47 patients with triple negative breast cancer (TNBC) enrolled onto different arms of a clinical trial with or without treatment of the PARP1 inhibitor olaparib. The dataset was split by individual patients pre- and post-treatment. As with CPC, RECODR was run on log-normalized counts subset to include genes expressed in at least 5% of cells and graph-networks were made with a Pearson correlation above 0.1. Node2Vec and Word2Vec were run with the same parameters as with CPC before models were aligned. Node scores were carried out for individual patients by comparing the post-treated graph to their corresponding pre-treated graph.

##### Medulloblastoma

We analyzed medulloblastoma pre- and post-treatment scRNAseq data from 9 patients. For all datasets, RECODR was run in the same way as with the CPC and TNBC data. Node scores were generated for recurrence samples compared to primary samples.

##### Identification of most variably expressed genes

Genes with the greatest variability in expression level were detected across medulloblastoma and TNBC samples using Scanpy in Python. First, the adata object was subset to data from patients pre- and post-treatment. Then, the *highy_variable_genes* function in Scanpy was used on each subset object with ‘n_top_genes’ set to 4500. The *get.obs_df* function from Scanpy was used to extract expression values for the 4500 most variable genes by patient. The mean expression of each gene was then calculated using the *mean* function of the expression dataframe. Finally, the mean absolute deviation was calculated using the *mad* function from the Pandas library.

## Supplementary Material

Supplemental information can be found online at https://doi.org/10.1016/j.ccell.2025.06.005.

Supplementary Figures

## Data Availability

No new materials were generated as part of this study. All scRNA-seq data generated as part of this study are deposited with reference GSE252995.

## Highlights

- Graph-embedding of cancer transcriptomes identifies drivers of treatment relapse
- Combination, relapse-mitigating treatment identified for choroid plexus carcinoma
- Gene context drift detectable in pre- and post-treatment samples of cancer patients
