# Supplementary material for: Gene context drift identifies drug targets to mitigate cancer treatment resistance
Source: Cancer Cell. Author manuscript; Available in PMC 2026 Aug 14. (PMC7619360; doi:10.1016/j.ccell.2025.06.005)
Supplement: Supplementary file 1 [file EMS216175-supplement-Supplementary_Figures.pdf]

Cancer Cell, Volume *43*

Supplemental information

Gene context drift identifies drug targets to mitigate cancer treatment resistance

Amir Jassim, Birgit V. Nimmervoll, Sabrina Terranova, Erica Nathan, Linda Hu, Jessica T. Taylor, Katherine E. Masih, Lisa Ruff, Matilde Duarte, Elizabeth Cooper, Gunjan Katyal, Melika Akhbari, Reuben J. Gilbertson, Jennifer C. Coleman, Joseph S. Toker, Colton Terhune, Gabriel Balmus, Stephen P. Jackson, Hailong Liu, Tao Jiang, Michael D. Taylor, Kui Hua, Jean E. Abraham, Mariella G. Filbin, Anthony Hill, Anarita Patrizi, Neil Dani, Aviv Regev, Maria K. Lehtinen, and Richard J. Gilbertson

FIGURE S1

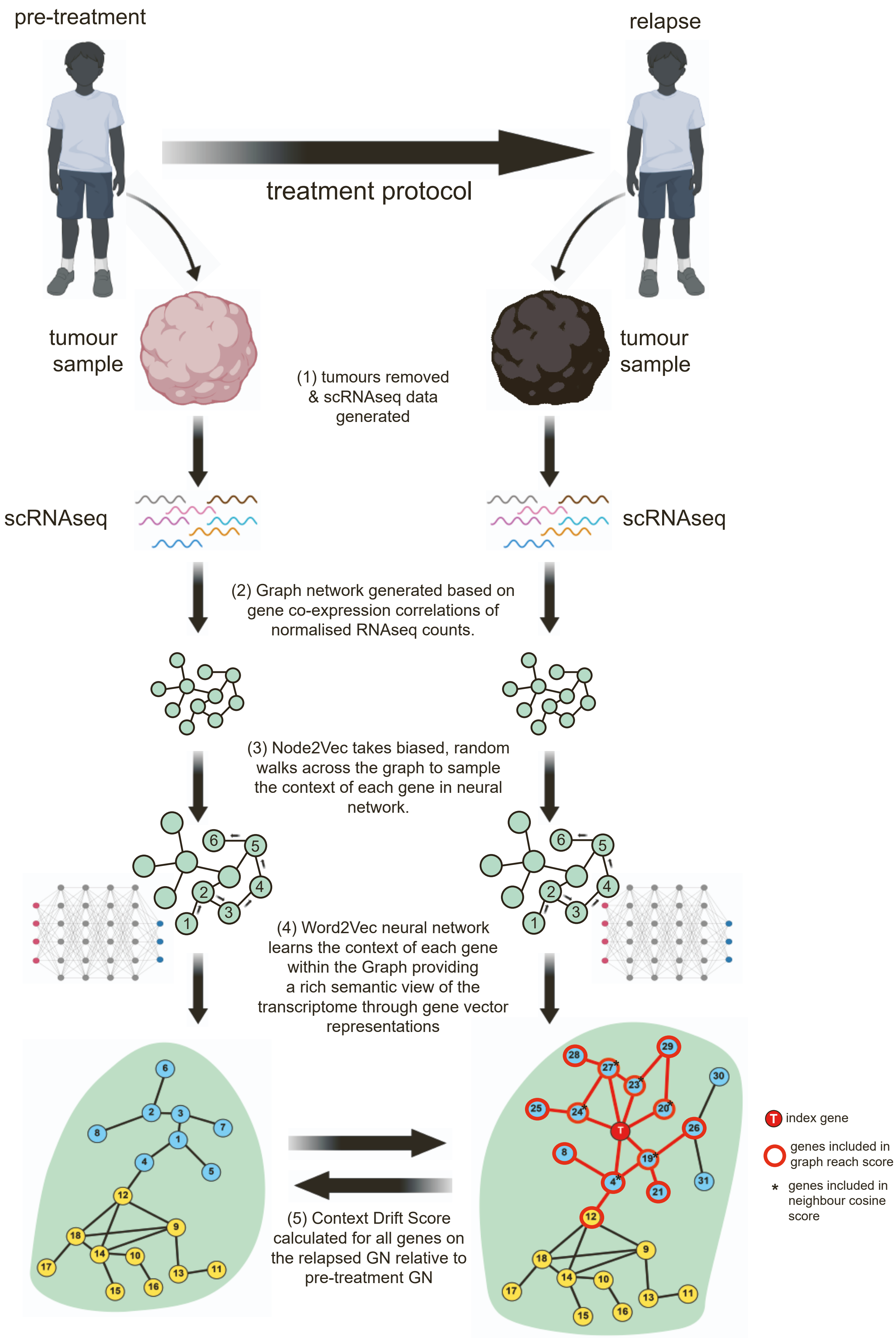

pre-treatment
relapse
treatment protocol
tumour sample
tumour sample
(1) tumours removed & scRNAseq data generated
scRNAseq
scRNAseq
(2) Graph network generated based on gene co-expression correlations of normalised RNAseq counts.
(3) Node2Vec takes biased, random walks across the graph to sample the context of each gene in neural network.
(4) Word2Vec neural network learns the context of each gene within the Graph providing a rich semantic view of the transcriptome through gene vector representations
(5) Context Drift Score calculated for all genes on the relapsed GN relative to pre-treatment GN
index gene
genes included in graph reach score
genes included in neighbour cosine score

Figure S1. The RECODR pipeline, related to Figure 1: (1) The pipeline starts with scRNAseq data taken from two conditions e.g., pre- and post-treatment. scRNAseq data are pre-processed and counts normalized. (2) Co-expression graph networks are constructed for each condition, co-clustering into communities tightly correlated, and therefore potentially functionally related, genes. (3) The Node2Vec algorithm enacted within RECODR takes random walks from every gene to generate a representation of the context around every gene in each condition. (4) These graph walks (context representation) are then trained by Word2Vec to generate embedding vector representations of every gene, learning the wider context of each gene in each condition. (5) The vectors for the same gene in each condition are then compared to generate an approximation of the extent to which their surrounding context has changed (context drift) before a final context drift score is generated to rank genes according to their degree of context change between conditions.

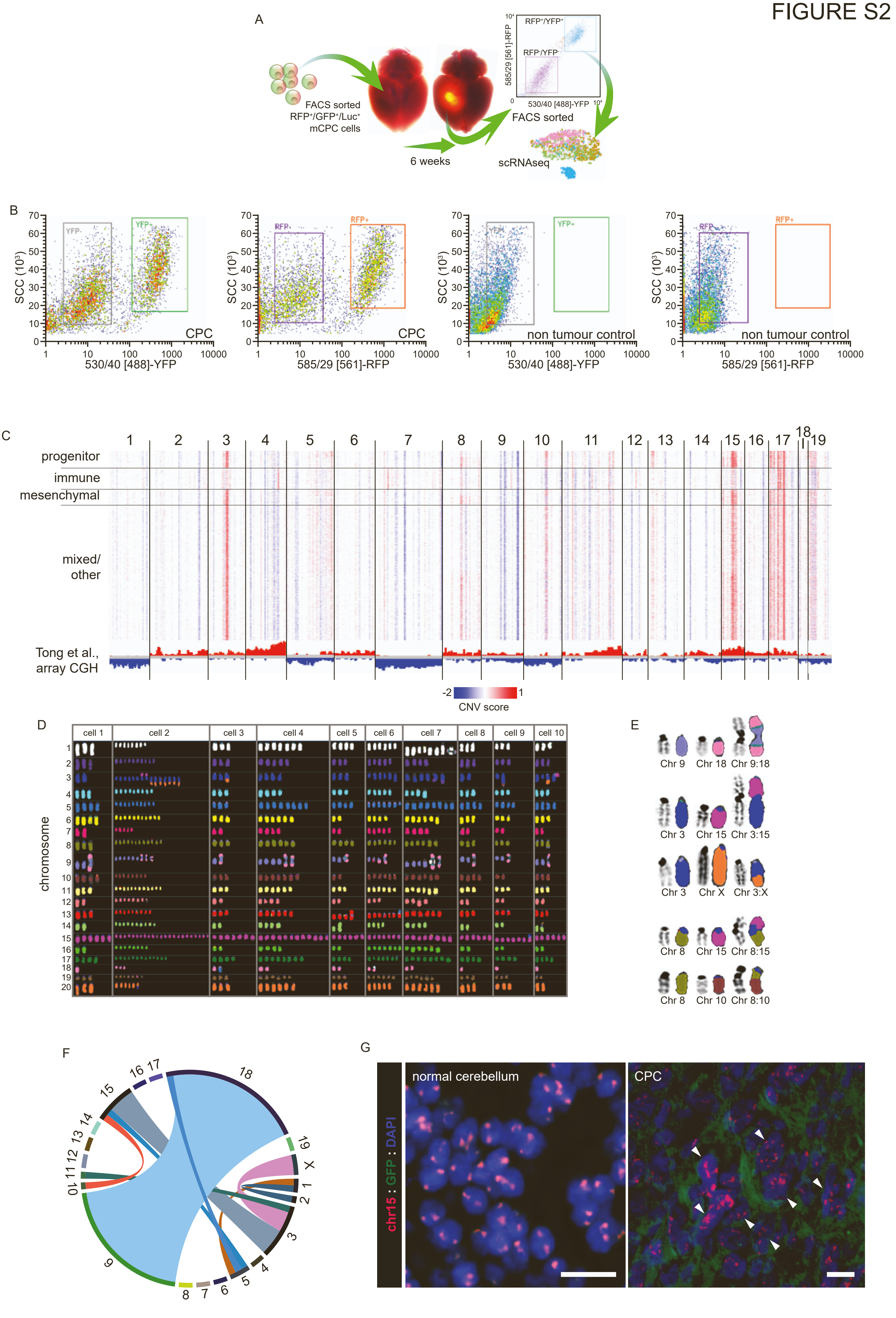
A
FACS sorted
RFP+/GFP+/Luc+
mCPC cells
6 weeks
RFP+/YFP+
RFP-/YFP-
585/29 [561]-RFP
530/40 [488]-YFP
FACS sorted
scRNAseq
B
SCC (10³)
YFP-
YFP+
RFP-
RFP+
CPC
non tumour control
530/40 [488]-YFP
585/29 [561]-RFP
C
progenitor
immune
mesenchymal
mixed/
other
Tong et al.,
array CGH
CNV score
D
chromosome
cell 1
cell 2
cell 3
cell 4
cell 5
cell 6
cell 7
cell 8
cell 9
cell 10
E
Chr 9
Chr 18
Chr 9:18
Chr 3
Chr 15
Chr 3:15
Chr X
Chr 3:X
Chr 8
Chr 8:15
Chr 10
Chr 8:10
F
G
normal cerebellum
CPC
chr15 : GFP : DAPI

Figure S2. Isolation and characterization of mouse CPC cells, related to Figure 1: A. Schematic depicting CPC implants and fluorescence activated cell sorting (FACS) for single-cell RNA sequencing (scRNAseq). B. Flow cytometry plots indicating gating of YFP+ (left) and RFP+ (right) CPC cells from tumour bearing (left) and non-tumour bearing (right) controls. C. Copy number variations (CNVs) inferred in scRNAseq profiles of each CPC cell. CNV scores at bottom are from reference 23. D. Spectral karyotype analysis of 10 individual CPC cells. E. Representative SKY images of intracellular translocations in CPC cells. F. CIRCOS plot summarizing intracellular chromosomal translocations detected by SKY in 30 CPC cells. G. Concurrent GFP immunofluorescence and chromosome 15 FISH of the normal cerebellum (left) and CPC (right). Scale bar=20μm.

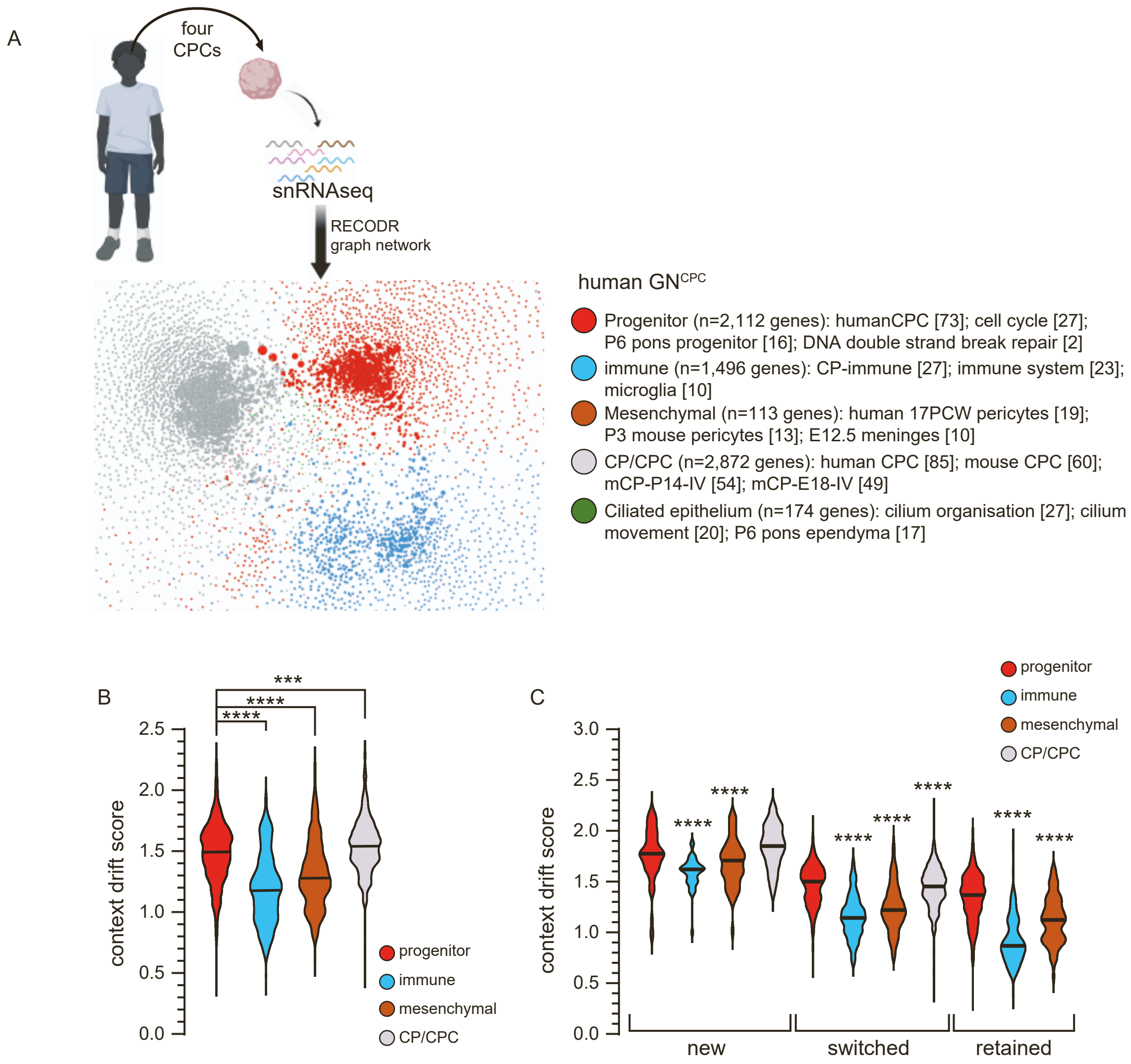

Figure S3. RECODR analysis of human and mouse CPC, related to Figure 1: A. GN of four independent human CPCs and communities and associated enriched gene sets. B. Violin plots of the context drift scores of genes in the indicated communities on the GNCPC (p values: ***=p<0.0005; ****=p<0.0005; Mann Whitney test). C. Violin plots of the context drift scores of retained, switched and new genes in the indicated communities on the GNCPC (p values: ****=p<0.0005; Mann Whitney).

FIGURE S4

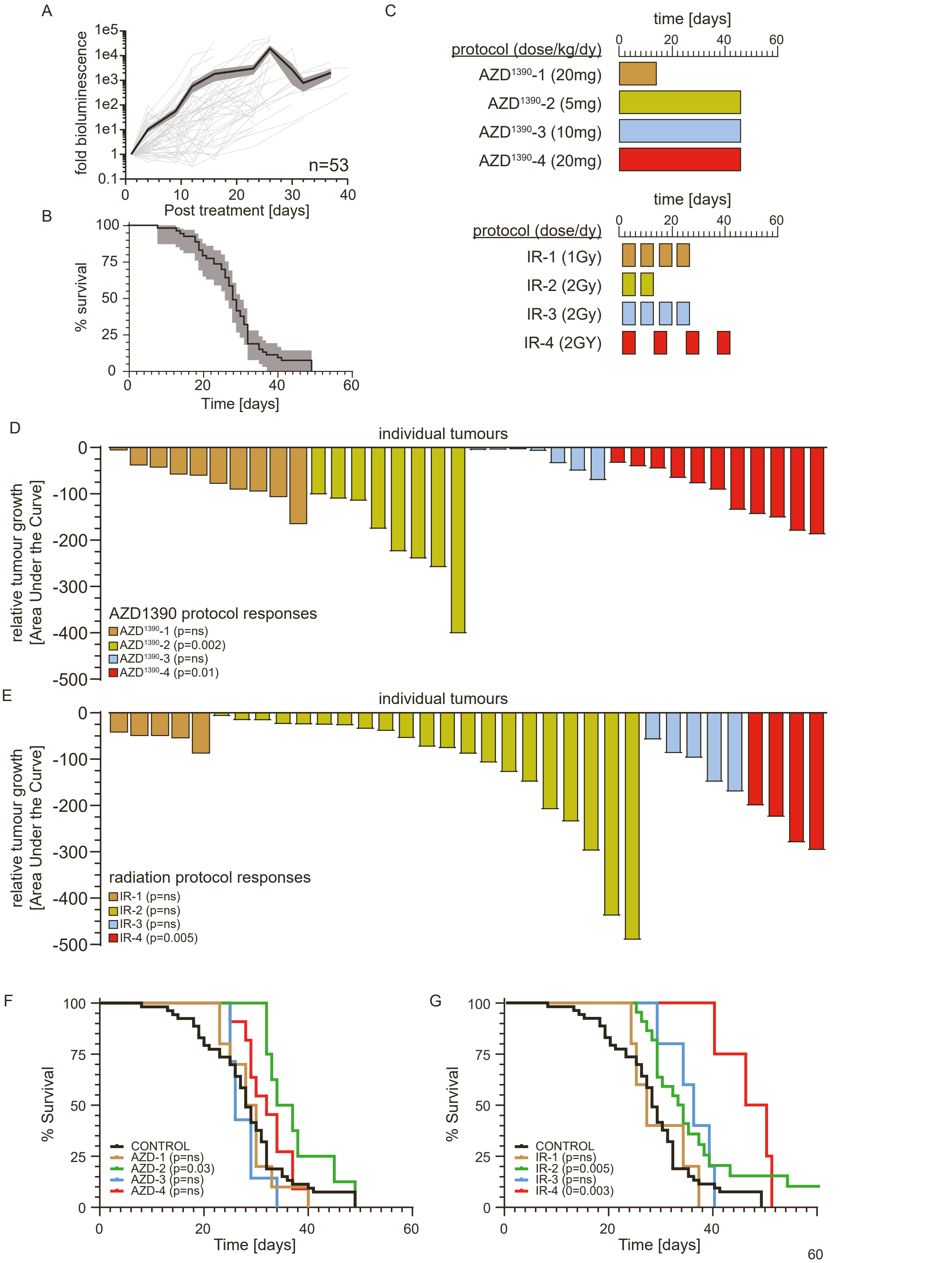

Figure S4. AZD1390 and radiation monotherapy regimens, related to Figure 2: Characterisation of control treated mouse tumour growth measured by bioluminescence, (A) and mouse survival (B) in 53 mice with CPC. C. Treatment protocols of AZD1390 (top) or radiation (bottom) monotherapy for mice with CPC (n=number of mice enrolled in each arm). Waterfall plots of AZD1390 (D) and radiation (E) treatments reporting tumour growth suppression of individual tumours over time during the indicated treatment. Bars report the total area under the curve of tumour growth over time measured by weekly tumour bioluminescence relative to control treated growth (p values record significant difference in growth relative to controls by Mann Whitney test). Survival curves of AZD1390 (F) and radiation (G) treatments reporting survival for the corresponding preclinical treatment protocols (p values report the Log Rank statistic relative to control treatment).

FIGURE S5

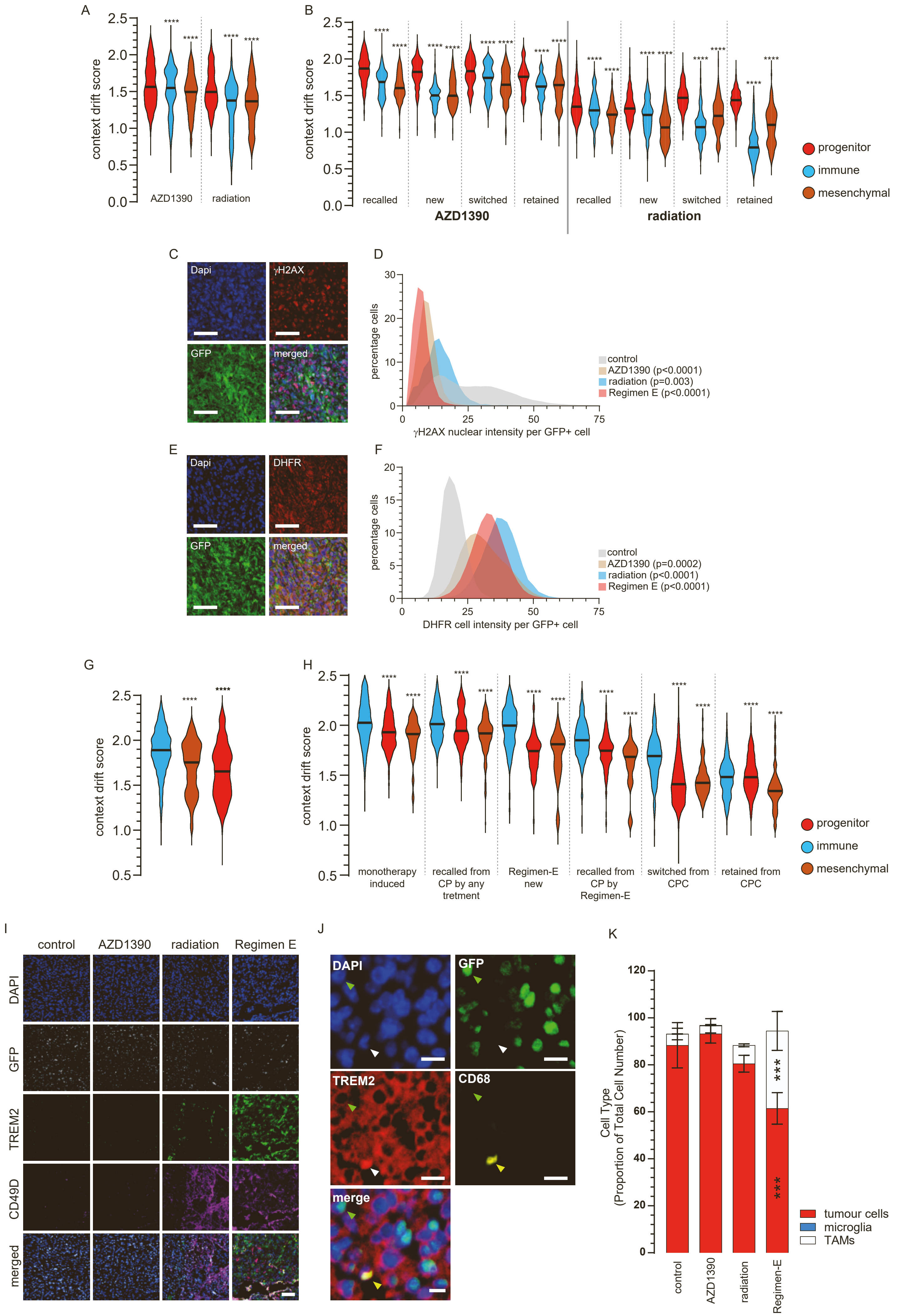
A
context drift score
AZD1390
radiation
B
recalled
new
switched
retained
AZD1390
radiation
progenitor
immune
mesenchymal
C
Dapi
γH2AX
GFP
merged
D
percentage cells
control
AZD1390 (p<0.0001)
radiation (p=0.003)
Regimen E (p<0.0001)
γH2AX nuclear intensity per GFP+ cell
E
Dapi
DHFR
GFP
merged
F
percentage cells
control
AZD1390 (p=0.0002)
radiation (p<0.0001)
Regimen E (p<0.0001)
DHFR cell intensity per GFP+ cell
G
context drift score
H
context drift score
monotherapy induced
recalled from CP by any tretment
Regimen-E new
recalled from CP by Regimen-E
switched from CPC
retained from CPC
progenitor
immune
mesenchymal
I
control
AZD1390
radiation
Regimen E
DAPI
GFP
TREM2
CD49D
merged
J
DAPI
GFP
TREM2
CD68
merge
K
Cell Type (Proportion of Total Cell Number)
control
AZD1390
radiation
Regimen-E
tumour cells
microglia
TAMs

Figure S5. Context drift scores in CPC cell populations during AZD1390 and radiation monotherapy and combination treatment resistance, related to Figure 3: A. Violin plots of the context drift scores of genes in the indicated communities on the GNAZD1390 and GNIR (p values: ****=p<0.0005; Mann Whitney; community key shown to right of panel B). B. Violin plots of the context drift scores of recalled, new, switched and retained genes in the indicated communities on the GNAZD1390 and GNIR (p values: ****=p<0.0005; Mann Whitney). C. Co-Immunofluorescence of γH2AX and GFP in exemplar AZD1390 resistant CPC (Scale bar=40μm). D. Frequency plot of γH2AX immunofluorescence intensity/GFP+ CPC cells across different conditions and control (untreated CPC). E. Co-Immunofluorescence of DHFR and GFP in exemplar radiation resistant CPC (Scale bar=40μm). F. Frequency plot of DHFR immunofluorescence intensity in GFP+ CPC cells across different conditions and control (untreated CPC). G. Violin plots of the context drift scores of genes in the indicated communities on the GNReg-E (p values: ****=p<0.0005; Mann Whitney; community key shown to right of panel H). H. Violin plots of the context drift scores of indicated gene types (see main text) in the indicated communities on the GNReg-E (p values: ****=p<0.0005; Mann Whitney). I. Co-immunofluorescence of GFP, TREM2, and vCD49D in CPCs treated as shown. Scale bar=50μm. J. Co-immunofluorescence of GFP, TREM2 and CD68 in Regimen-E relapsed CPC showing GFP+ CPC cells and GFP-/CD68+ TAM. Scale bar=20μm. K. Mean proportion (+standard deviation) of tumour associated macrophages (TAMs; CD45+/CD68+/P2Y12+/CD49+), microglia (CD45+/CD68+/P2Y12+/CD49-) and GFP+ tumour cells across all conditions. ***, p<0.0005 Mann Whitney.

FIGURE S6

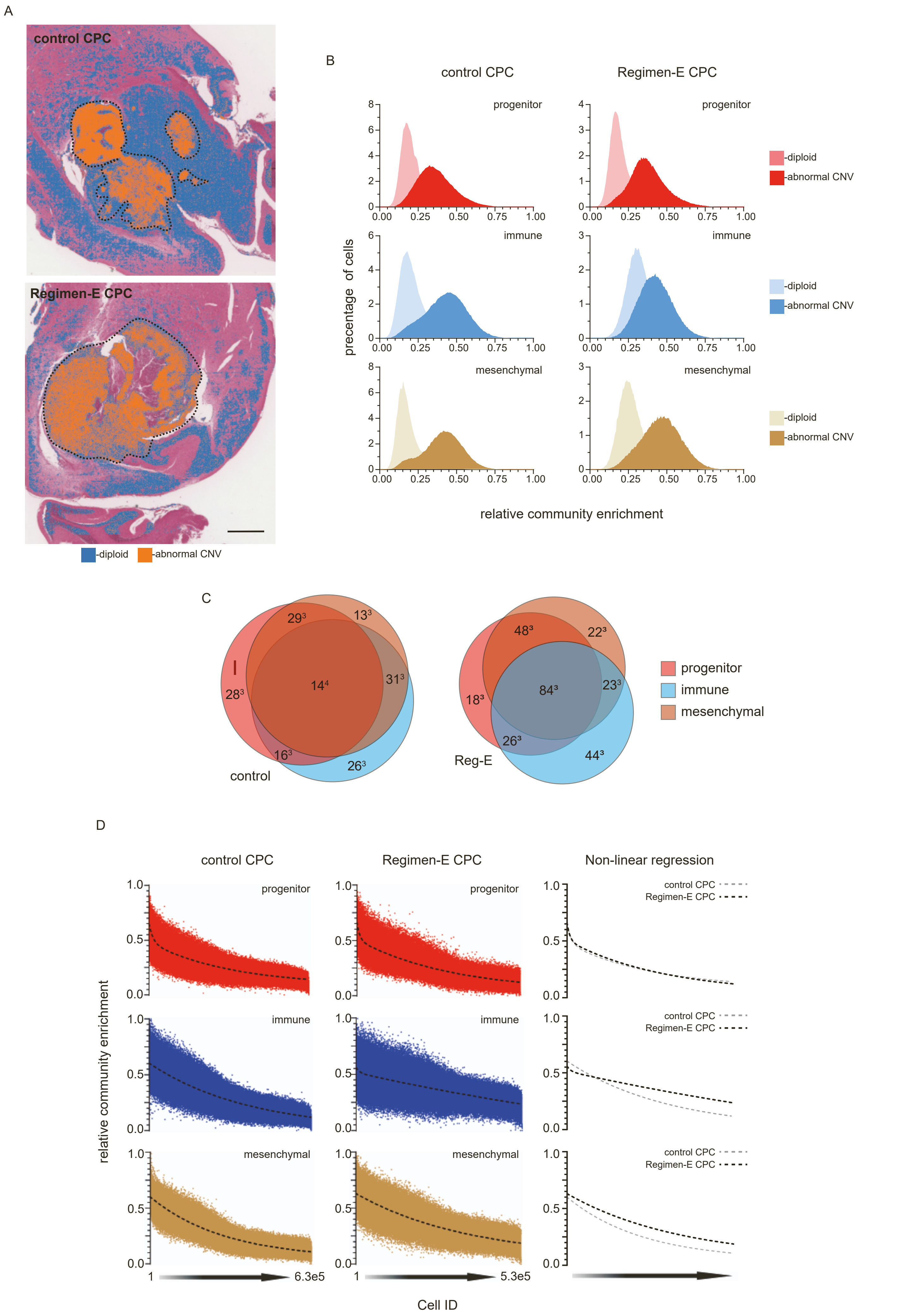
A
control CPC
Regimen-E CPC
-diploid
-abnormal CNV
B
control CPC
Regimen-E CPC
progenitor
immune
mesenchymal
precentage of cells
-diploid
-abnormal CNV
relative community enrichment
C
progenitor
immune
mesenchymal
29^3
13^3
28^3
14^4
31^3
16^3
26^3
control
48^3
22^3
18^3
84^3
23^3
26^3
44^3
Reg-E
D
control CPC
Regimen-E CPC
Non-linear regression
progenitor
immune
mesenchymal
control CPC
Regimen-E CPC
relative community enrichment
1
6.3e5
5.3e5
Cell ID

Figure S6. Visium HD spatial transcriptomic analysis of control treated and Regimen-E relapsed CPC, related to Figure 5: A. Inferred copy number abnormalities inferred as normal diploid and tumour cells with abnormal chromosome copy number variance (CNV) in untreated CPC (top) and Regimen-E relapsed CPC (bottom; scale bar=1mm). Hashed line outlines tumour in each. B. Frequency plots of relative enrichment of expression of the indicated community in inferred normal and tumour cells in untreated and Regimen-E relapsed CPC detected by Visium HD. C. Venn diagrams of Visium HD bins enriched for the indicated communities in control and Regimen-E resistant CPC. D. Graphs left report the ranked relative enrichment of expression of progenitor (top), immune (middle) and mesenchymal (bottom) community genes in all 8μmx8μm bins detected by Visium HD in sections of control CPC. Middle graphs report the same in sections of Regimen-E relapsed CPC. Right graphs report the non-linear regression curves of the corresponding left and middle graphs.

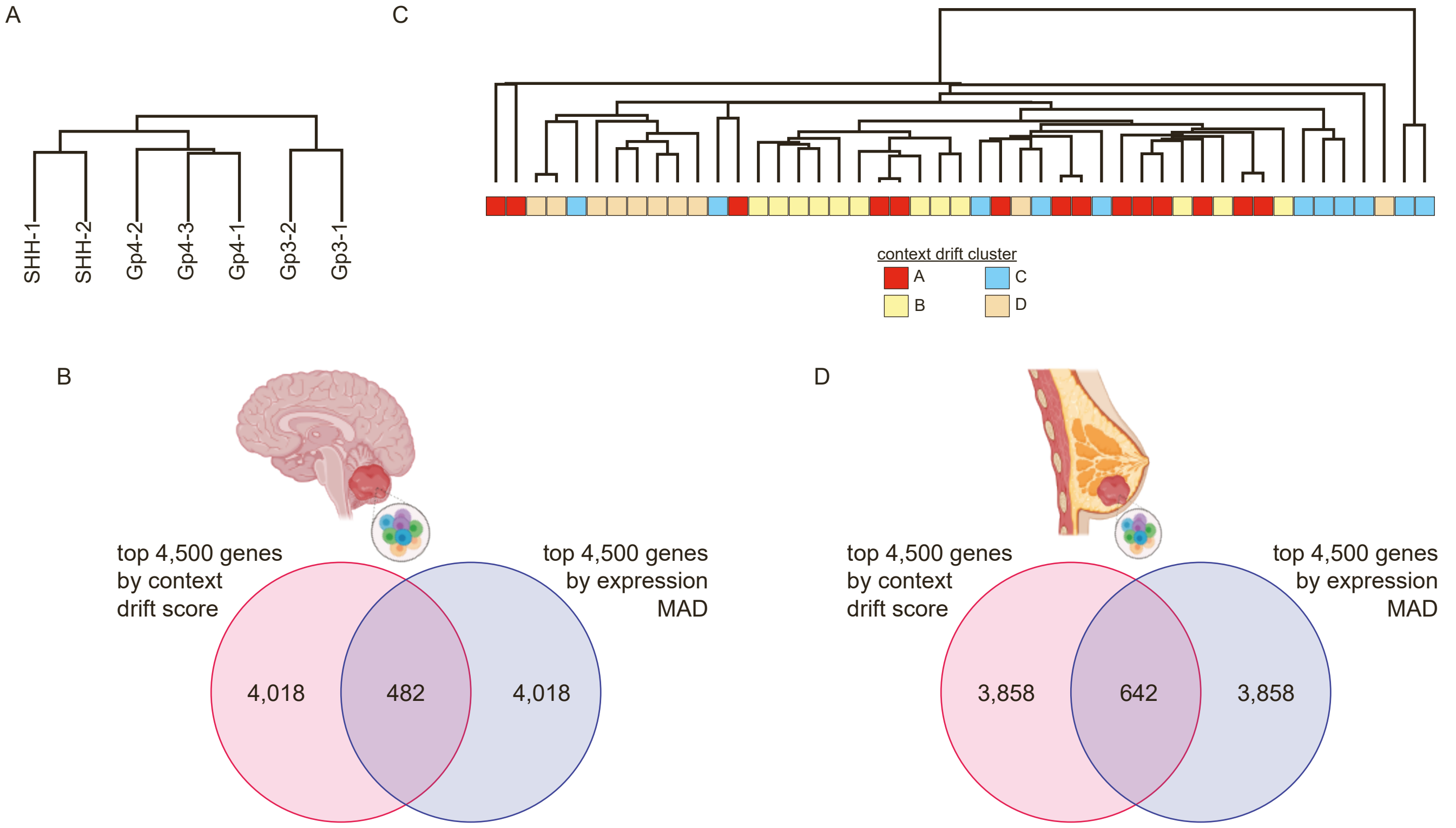

Figure S7. Comparison of gene expression levels and context level drift scores among patients with medulloblastoma or triple negative breast cancer, related to Figure 7: A. Unsupervised hierarchical clustering by Euclidean distance of the 4,500 genes with the most variable expression across medulloblastoma sc/snRNAseq profiles analysed in Figure 6 converted to bulk sequence (see STAR Methods). Expression level variance was determined by mean absolute deviation (MAD) score. B. Venn diagrams report the overlap in the top 4,500 genes with the highest context drift scores or MAD gene expression scores across medulloblastomas. C. Unsupervised hierarchical clustering by Euclidean distance of the 4,500 genes with the most variable expression across triple negative breast cancer snRNAseq profiles analysed in Figure 6 converted to bulk sequence (see STAR Methods). Expression level variance was determined by MAD score. D. Venn diagrams report the overlap in the top 4,500 genes with the highest context drift scores or MAD gene expression scores across triple negative breast cancers
